# Scalable Defect Engineering of Pt_3_Te_4_ Nanosheets Activates an Electro-Switchable and Termination-Dependent PtO_2_ Skin for Low-Overpotential Hydrogen Evolution

**DOI:** 10.1021/acsami.5c18460

**Published:** 2026-01-27

**Authors:** Tsotne Dadiani, Gianluca D’Olimpio, Loreta Tamasauskaite-Tamasiunaite, Stefano Zenone, Chia-Nung Kuo, Matteo Amati, Zygmunt Milosz, Luca Gregoratti, Tomáš Hrbek, Miquel Gamón Rodríguez, Marian Cosmin Istrate, Chin Shan Lue, Yevheniia Lobko, Corneliu Ghica, Eugenijus Norkus, Yong-Wei Zhang, Anna Cupolillo, Danil W. Boukhvalov, Antonio Politano

**Affiliations:** † Department of Physical and Chemical Sciences, University of L’Aquila, via Vetoio, 67100 L’Aquila (AQ), Italy; ‡ Department of Catalysis, Center for Physical Sciences and Technology, Sauletekio Ave. 3, LT-10257 Vilnius, Lithuania; § Department of Applied Science and Technology, Polytechnic University of Turin, Corso Castelfidardo, 39, 10129 Turin, Italy; ∥ Program on Key Materials, Academy of Innovative Semiconductor and Sustainable Manufacturing (AISSM), 34912 National Cheng Kung University, Tainan 70101, Taiwan; ⊥ Department of Physics, National Cheng Kung University, Tainan 70101, Taiwan; # Taiwan Consortium of Emergent Crystalline Materials (TCECM), National Science and Technology Council, Taipei 10601, Taiwan; ∇ Elettra—Sincrotrone Trieste SCpA, AREA Science Park, Strada Statale 14 km 163.5, 34149 Trieste, Italy; ○ Department of Surface and Plasma Science, Faculty of Mathematics and Physics, 37740 Charles University, V Holešovičkách 2, 18000 Prague, Czech Republic; ◆ 198818 National Institute of Materials Physics, Atomistilor 405A, 077125 Magurele, Romania; ¶ Institute of High Performance Computing (IHPC), Agency for Science, Technology and Research (A*STAR), Singapore 138632, Republic of Singapore; †† Department of Physics, University of Calabria, Via P. Bucci cubo 31/C, Rende, Cosenza 87036, Italy; ‡‡ College of Science, Institute of Materials Physics and Chemistry, 74584 Nanjing Forestry University, Nanjing 210037, P. R. China; §§ Institute of Physics and Technology, Satbayev University, Ibragimov str. 11, Almaty 050032, Kazakhstan

**Keywords:** platinum telluride, liquid-phase exfoliation, defect engineering, hydrogen evolution reaction, electroswitchable oxide skin, termination-dependent catalysis, scanning photoemission microscopy, operando XPS

## Abstract

Topological materials are promising electrocatalysts for the hydrogen evolution reaction (HER) because of their protected electronic states and exceptional carrier mobility. Among them, the topological metal Pt_3_Te_4_ (mitrofanovite) exhibits low Tafel slopes in the nanocrystals. Realizing this potential in scalable catalyst systems requires nanoscale texturing coupled with precise control of the surface chemistry under operating conditions. Herein, we demonstrate that hydrogen peroxide (H_2_O_2_)-assisted liquid-phase exfoliation (LPE) of bulk Pt_3_Te_4_ yields nanoporous nanosheets that retain their metallic character and are chemically preconditioned to develop a bias-controlled PtO_2_ skin that governs the catalytic activity. Crucially, spectromicroscopy resolves termination-selective oxidation: PtO_2_ forms exclusively on PtTe_2_-like terminations, whereas Pt_2_Te_2_ terminations remain metallic. Operando ambient-pressure X-ray photoelectron spectroscopy (AP-XPS) in an electrochemical cell revealed the bias-dependent emergence of surface oxide phases in H_2_O_2_-treated nanosheets. The joint effect of the higher accessible site density imparted by nanoporosity and the emergence of a bias-controlled PtO_2_/PtTe_2_-terminated Pt_3_Te_4_ surface junction rationalizes the improved catalytic activity: the overpotential at 10 mA cm^–2^ decreases by ∼30% (from 113.1 to 78.7 mV), while the exchange current density more than triples (from 0.106 to 0.347 mA cm^–2^), all with an unchanged Tafel slope (∼53 mV dec^–1^) and sustained stability over 50 h in acid. By combining a single scalable top-down step with operando proof that the catalytically active oxide is switched on by bias rather than being a static passivation layer, this study establishes a precise interface-engineering principle for Pt_3_Te_4_ nanosheets and a practical path to efficient, scalable HER catalysts based on nanosheets of topological metals.

## Graphical abstract

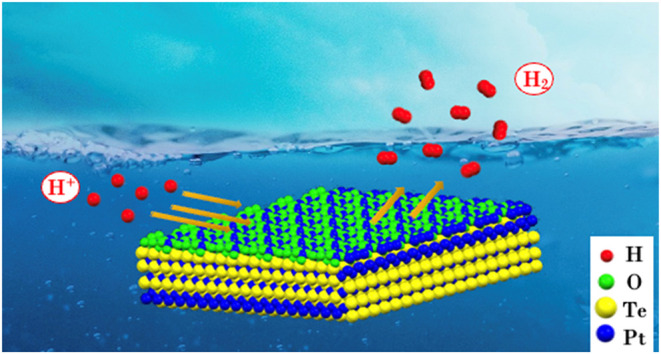

## 1 Introduction

Van der Waals (vdW) materials have opened new pathways for catalysis because of their intrinsically high surface-to-volume ratios, which maximize the density of active sites.^1^ In particular, transition-metal dichalcogenides (TMDs), such as MoS_2_ ^2,3^ and WS_2_,^2,4,5^ have been extensively used as catalysts for the HER, as they provide complementary properties compared to standard transition-metal catalysts.^6–8^ Nevertheless, two-dimensional (2D) materials still face notable challenges related to their stability under operational conditions.^9^ Therefore, it is highly desirable to develop new strategies to preserve the structural integrity and catalytic activity of 2D materials under realistic electrochemical conditions,^10^ where environmental factors such as pH and applied potential can induce decomposition or structural modifications of the catalyst.^11,12^ Stability problems arise because the active sites, which are typically found at the edges of nanosheets, are prone to degradation.^13^ Accordingly, vdW materials exhibiting topological properties are promising candidates for electrocatalytic applications,^14–16^ because they display topologically protected electronic states that are immune to scattering from nonmagnetic impurities and defects.^16,17^ This robustness makes topological materials particularly appealing for applications that require durability, such as electrocatalytic electrodes.^15^ Moreover, the inherently high mobility of charge carriers in topological materials plays a crucial role in enhancing the kinetics of catalytic processes.^18^

Among topological vdW materials, mitrofanovite (Pt_3_Te_4_)^19^ has recently attracted attention as an HER electrocatalyst, with nanocrystals showing overpotentials of 280–370 mV at 10 mA cm^–2^ ^20^ and a Tafel slope of ∼33 mV dec^–1^.^21^ The presence of topologically protected surface states^19^ is beneficial for durability under harsh conditions typical of the HER. Additionally, the weak interlayer vdW bonds enable exfoliation into 2D nanosheets, thus increasing the surface area and number of active sites for catalytic processes.

Optimizing the number of active sites on the catalyst surface is critical for improving its efficiency.^22^ Several strategies have been studied to increase the catalytic activity of 2D materials,^12,23–26^ many of which face obstacles in terms of scalability, cost-effectiveness, and reproducibility when applied on a larger scale.

One common method to increase the number of active sites in 2D materials is chemical doping with heteroatoms,^27^ which also introduces dopant-derived electronic states that favor catalytic reactions,^28^ particularly at the edges of 2D nanosheets compared to their basal planes.^28^ However, chemical doping often requires precise control of the doping concentration and distribution, which can be difficult to achieve uniformly across large-scale production.^29^ Moreover, the incorporation of dopants may occasionally result in instability, especially under harsh conditions such as in the HER, where extended contact with electrolytes can induce dopant leaching, consequently diminishing the catalytic activity over time.^30^

Another method involves increasing the number of active sites by forming defects in 2D nanosheets.^31–33^ However, the controlled implantation of such defects is challenging and often requires complex techniques such as plasma treatment,^34^ ion irradiation,^34^ and electron irradiation.^34^ These techniques are expensive and difficult to scale up, limiting their practical application in large-scale catalytic systems. Furthermore, the reproducibility of defect engineering is another significant obstacle, as the extent and nature of the defects can vary significantly, leading to inconsistent catalytic performance.^35^

The exfoliation of bulk crystals produces nanosheets with high surface areas and numerous active sites.^36,37^ Although mechanical exfoliation can produce high-quality nanosheets, it is not scalable and is typically limited to laboratory experiments.^35^ In contrast, LPE is scalable and can produce a large number of nanosheets.^38^ However, controlling the defect density is challenging.^39^ Typically, the edges of nanosheets act as the main active sites, whereas the basal plane remains relatively inert.^12,40–42^ However, such edge-dominated reactivity is insufficient for realistic technology transfer.^43^ Accordingly, it is crucial to develop methods that introduce defects or active sites directly onto the basal plane to make the entire surface area of the nanosheets catalytically or functionally active,^25^ rather than being limited to edge sites.

These constraints point to a complementary strategy that does not merely pre-engineer more sites ex situ but also identifies and programs the surface that actually exists under operation. Operando surface science tools should be used to interrogate and identify the active interface, which can be transformed under bias, because interfacial fields and charge accumulation can reversibly create a catalytically competent state.^44^ Accordingly, studying the catalytic surface under an applied bias is indispensable because the polarized electrode–electrolyte interface constitutes a distinct thermodynamic and electronic state that does not exist in an open circuit.^45^ The electrode potential shifts the Fermi level relative to the solution redox couples, fixes the surface charge through the potential of zero charge, and establishes intense interfacial fields that reorder adsorption equilibria, alter oxidation states, and stabilize metastable surface terminations or ultrathin oxide skins that are absent ex situ.^44,46^ Any ex situ snapshot, regardless of how carefully prepared, collapses the double layer and relaxes field-stabilized configurations, severing the causal link between polarization and chemistry. Only operando measurements at controlled potentials recover that link by tracking the evolution of bonding and short-range order in real time as the current passes.^47,48^ Thus, bias-driven surface transformations represent an emerging hot topic at the boundary between surface science and electrocatalysis, challenging the traditional view of catalyst surfaces as static under reaction conditions.^48,49^ Recent operando and in situ studies have revealed that applied potentials can dynamically and reversibly alter the surface oxidation states, lattice terminations, active-site distributions, and surface stoichiometry,^50,51^ thus affecting the catalytic performance.

Here, we implemented hydrogen peroxide (H_2_O_2_)-assisted LPE to engineer nanoporous Pt_3_Te_4_ nanosheets, combining morphological control with operando AP-XPS in an electrochemical cell. The use of H_2_O_2_ as a mild oxidant enabled gradual and tunable etching of the Pt_3_Te_4_ basal planes, resulting in the formation of nanoscale pores and disordered edges, the intensity of which scaled with the H_2_O_2_ concentration. This process increased the density of the catalytically active sites without compromising the crystallinity of flakes.

Because its unit cell contains two nonequivalent Pt–Te slabs, Pt_3_Te_4_ cleaves into Pt_2_Te_2_- and PtTe_2_-terminated surfaces.^52^ The nondegenerate surface electronic states at each termination modulate the adsorption energies and interfacial charge transfer, thereby governing their chemical reactivity.^52^ Spatially resolved and surface-sensitive XPS measurements revealed that oxidative treatment selectively affected the PtTe_2_ surface termination, promoting the in situ formation of catalytically active PtO_2_ species at moderate cathodic potentials, as revealed by operando AP-XPS in a dedicated electrochemical half-cell configuration. This behavior was absent in the pristine samples, where PtO_2_ was formed only at a more negative bias. Meanwhile, Te atoms show dual behavior: although generally stable, they undergo partial oxidation and even hydrogenation at under-coordinated sites during the HER. Therefore, our results highlight that the applied bias can act as an active lever to program catalyst surfaces in real time, thereby opening new possibilities for the design of dynamic, self-adapting electrocatalysts.

The catalytic performance of the H_2_O_2_-treated nanosheets reflects their surface reactivity. In particular, nanosheets exfoliated with 0.3% H_2_O_2_ reached a current density of 10 mA cm^–2^ at a remarkably low overpotential of 78.7 mV, with excellent long-term stability over 50 h. Therefore, the H_2_O_2_-treated Pt_3_Te_4_ nanosheets exhibited a combination of surface porosity, catalytically active nanoscale surface oxide species, and high electrode durability.

## 2 Results and Discussion

### 2.1 Morphological Investigation

Pt_3_Te_4_ flakes were obtained via LPE from bulk crystals in the absence and presence of hydrogen peroxide (H_2_O_2_) as an oxidizing agent. The morphological modifications induced by H_2_O_2_ treatment were analyzed using scanning electron microscopy (SEM), atomic force microscopy (AFM) (Figure 1), and transmission electron microscopy (TEM, Figure 2).

**Figure 1:**
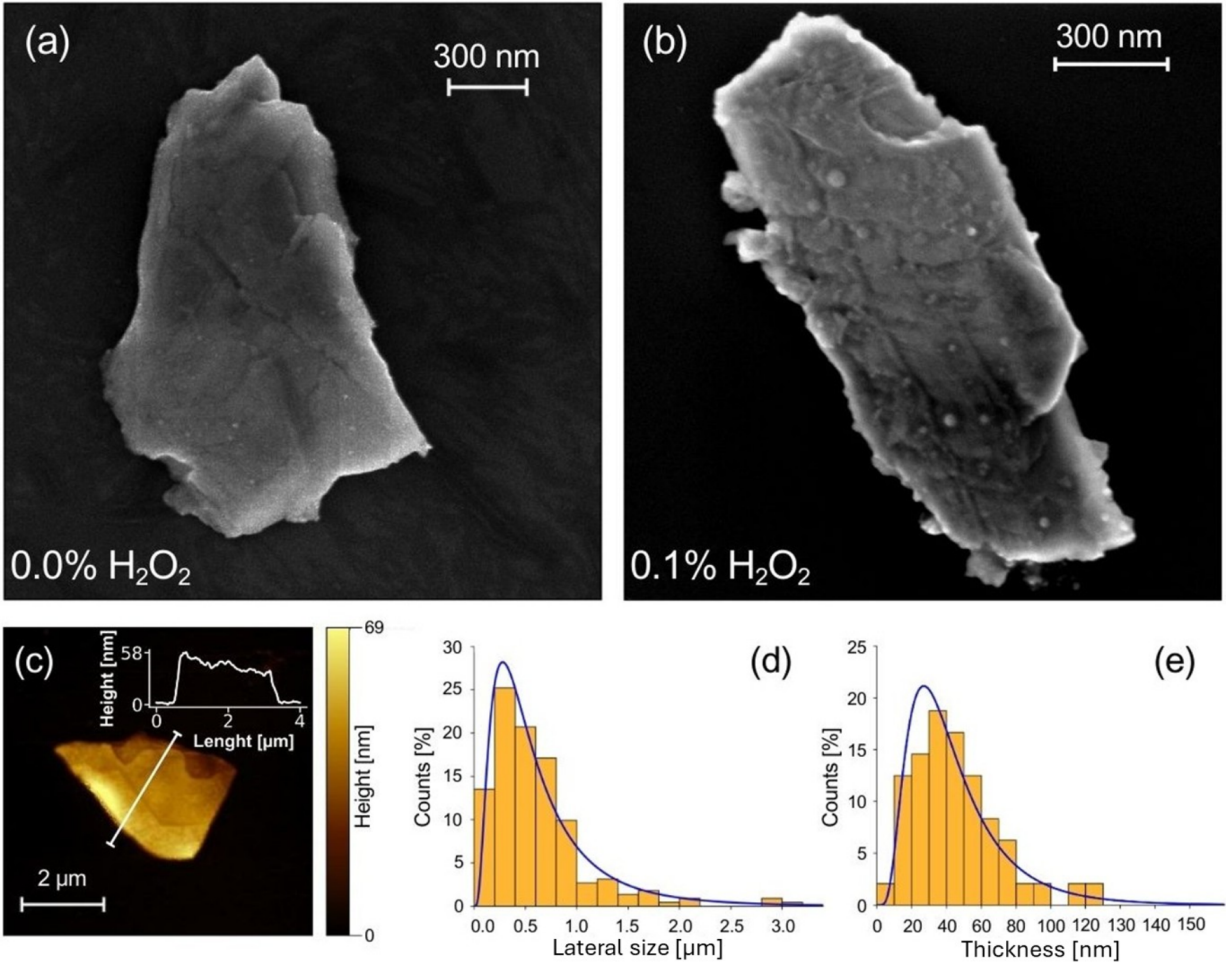
SEM images of Pt_3_Te_4_ flakes obtained using LPE. (a) Flakes exfoliated without H_2_O_2_ displayed a relatively smooth surface. (b) Flakes exfoliated using 0.1% H_2_O_2_-assisted LPE with nanostructured surface. The flakes shown are not identical but represent general morphological changes resulting from H_2_O_2_ treatment. (c) AFM image of a selected Pt_3_Te_4_ flake. The corresponding line profile acquired along the white line is shown in the inset. (d) Lateral size and (e) thickness distributions of Pt_3_Te_4_ flakes obtained through LPE obtained.

**Figure 2:**
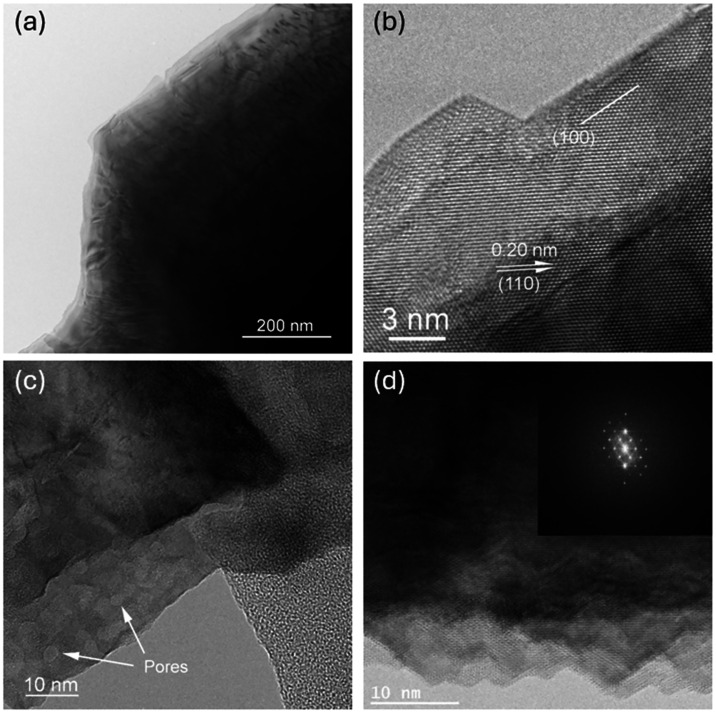
(a) and (b) HR-TEM images of pristine Pt_3_Te_4_ in the [001] orientation, showing the step-like thickness near the flake border due to exfoliation on the (001) planes. (c) and (d) Fragments of a typical Pt_3_Te_4_ flake obtained via LPE after treatment with 0.2% H_2_O_2_. The formation of pores and rugged, thin edges in the crystal grains was observed.

AFM analysis was performed to assess the thickness variations of the exfoliated Pt_3_Te_4_ flakes before and after the H_2_O_2_ treatment. Statistical analysis of the height profiles indicated that the average thickness was ∼ 50 nm for both untreated and modified flakes. The lateral size distribution probed by SEM (Figure 1d) and TEM showed an average side length of ∼500 nm with negligible differences. Thus, it can be inferred that oxidative exfoliation did not induce significant alterations in the overall size of flakes. However, the surface morphology was clearly affected: pristine flakes exhibited relatively well-defined and flat surfaces (SEM image in Figure 1a), whereas the flakes exfoliated in the presence of H_2_O_2_ (Figure 1b in the case of 0.1% H_2_O_2_) displayed a rougher texture with the appearance of surface nanostructures.

These results were supported by TEM analysis (Figure 2), which offered deep insights into the effects of H_2_O_2_ treatment at the nanoscale. The pristine Pt_3_Te_4_ nanosheets displayed highly crystalline structures reaching the grain boundaries, characterized by step-like patterns due to exfoliation along the (001) planes and edges aligned with the (100) planes (Figure 2b). No indication of disorder or amorphization was observed (Figure S1 in the Supporting Information).

In contrast, HR-TEM images of the Pt_3_Te_4_ nanosheets obtained by LPE in IPA assisted by 0.1% H_2_O_2_ showed the appearance of a thin (2–5 nm) disordered or amorphous layer at the flake edge, along with planar defects, indicating the onset of surface degradation due to H_2_O_2_ (see Figure S3 in the Supporting Information). For the Pt_3_Te_4_ nanosheets obtained via LPE with 0.2% H_2_O_2_, the TEM images revealed even more pronounced effects: the flake edges became porous and irregular, and shallow surface cavities formed in thinner regions (Figure 2c). However, the small-area electron diffraction (SAED) pattern clearly indicated that the atomic structure of the bulk crystal remained unaffected (Figure 2d). Notably, oxygen was not detected within the flakes of any of the H_2_O_2_-treated Pt_3_Te_4_ nanosheets using energy-dispersive X-ray spectroscopy (EDS, Figures S2, S4, and S6 (Supporting Information)), suggesting that the chemical treatment led to surface morphology changes without significant oxygen incorporation into the bulk of the flakes. Therefore, within the explored H_2_O_2_ concentration window (0.1–0.3%), the impact of the oxidative treatment becomes progressively more pronounced, evolving from a mainly edge-localized disordered/amorphized layer at 0.1% to the clear appearance of pore-like surface features and markedly rugged edges already at 0.2% (Figure 2c,d).

### 2.2 Surface Chemical Reactivity

Spatially resolved photoemission electron microscopy (SPEM) measurements were performed to investigate the evolution of the surface terminations of Pt_3_Te_4_ during exfoliation under oxidative conditions. SPEM combines XPS with high spatial resolution, enabling the visualization of chemical heterogeneities at the surface with a resolution of 120 nm. This spatially resolved analysis is essential for understanding the relationship between the surface terminations of Pt_3_Te_4_ ^52^ and the catalytic activity, particularly when exploring H_2_O_2_-assisted LPE treatment.

Figure 3 shows the images obtained using SPEM at a kinetic energy corresponding to the Pt-4f core level. Figure 3a shows the as-cleaved Pt_3_Te_4_ sample, and Figure 3b shows the same surface after treatment with 0.1% H_2_O_2_. In the as-cleaved sample, a distinct contrast between the bright and dark regions was observed, reflecting variations in the peak intensity ratio of Pt_2_Te_2_/PtTe_2_. This contrast provides direct evidence of the heterogeneous nature of the Pt_3_Te_4_ surface, where two distinct chemical terminations coexist^52^ on the micrometer scale. To understand the origin of this contrast, we acquired spatially resolved XPS spectra from the specific areas marked by colored squares in Figure 3a,b. The Pt-4f core-level spectra (Figure 3c) revealed two distinct components corresponding to the different surface terminations of Pt_3_Te_4_. In the bright regions, the main peak appeared at a binding energy (BE) of 71.5 eV, which is assigned to the Pt_2_Te_2_ termination and reflects a more electron-rich environment. In contrast, the dark regions show an additional component at approximately 72.4 eV, which is associated with the PtTe_2_ termination. This spectral splitting of the Pt-4f_7/2_ peak indicates the coexistence of two electronic environments: the lower-BE component corresponds to Pt sites with a higher electron density (Pt_2_Te_2_), whereas the higher-BE component reflects Pt sites with a reduced electron density (PtTe_2_).^52^

**Figure 3:**
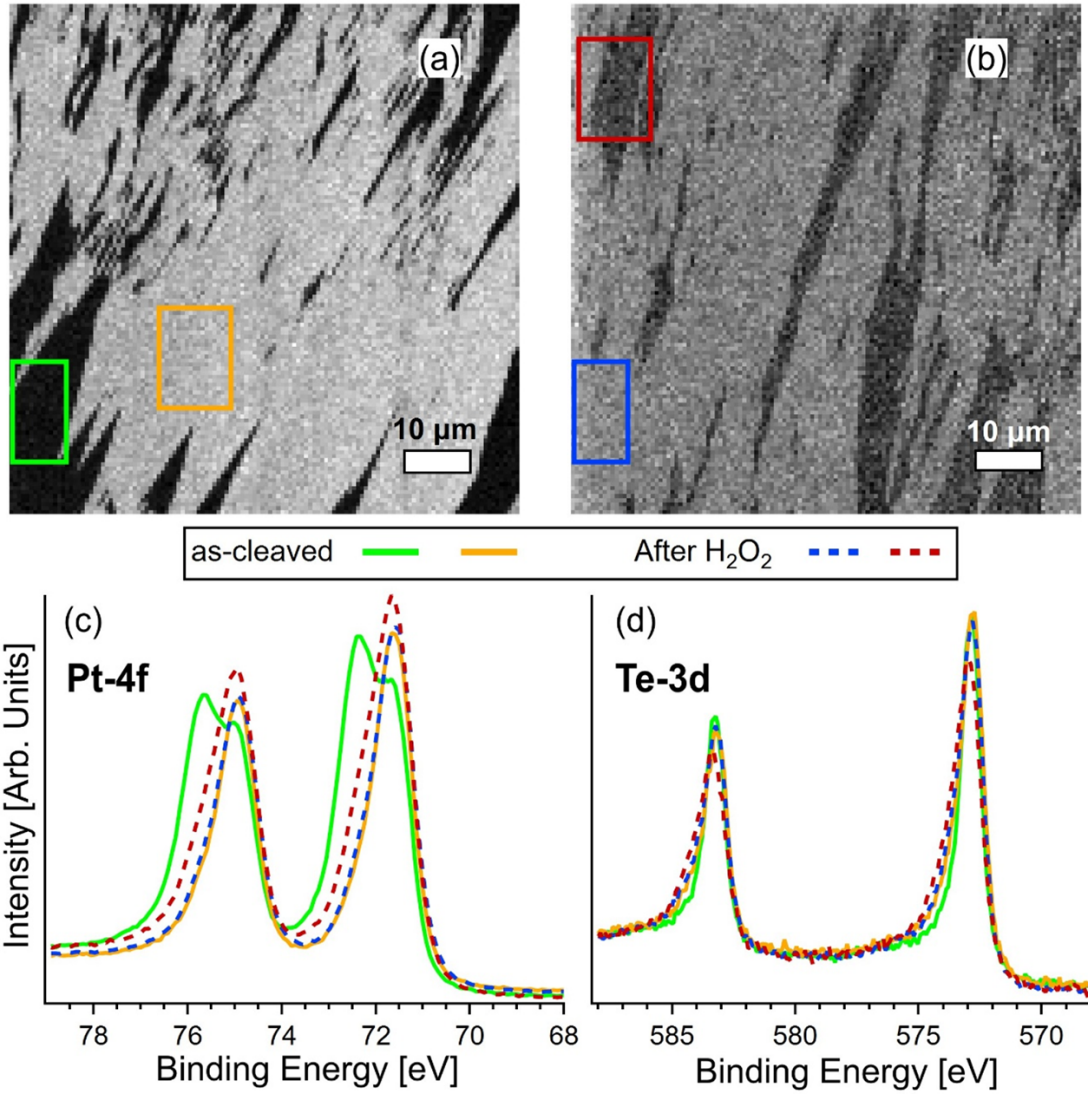
(a, b) SPEM images taken at Pt-4f kinetic energy. (a) As-cleaved surface and (b) surface after treatment with 0.1% H_2_O_2_. (c, d) XPS spectra extracted from selected points in the images before and after treatment.

Following oxidative treatment with 0.1% H_2_O_2_ (Figure 3b), we observed the redistribution of these surface terminations. After the H_2_O_2_ treatment, the Pt-4f spectra no longer exhibited a double-component structure. Only the lower-BE peak at 71.5 eV remained, indicating the complete disappearance of the higher-BE component associated with the PtTe_2_ termination. This result confirms that the oxidative environment selectively affects the PtTe_2_ termination, producing an oxidized phase, whereas the more stable and electron-rich Pt_2_Te_2_ termination remains intact.

Furthermore, the Te-3d core-level spectra (Figure 3d) remained unchanged following exposure to H_2_O_2_, thereby confirming the chemical stability of Te, regardless of the termination type. This finding aligns with the observation that the main impact of the differences between the Pt_2_Te_2_ and PtTe_2_ terminations is on the Pt electronic environment, whereas the Te atoms maintain their oxidation state regardless of variations in surface chemistry. This selective modification emphasizes the importance of understanding the termination-dependent chemical reactivity of Pt_3_Te_4_, which is crucial for optimizing its catalytic performance, particularly in applications such as the HER.

Having experimentally confirmed the modification of surface terminations upon H_2_O_2_ treatment, we modeled the energetics of these modifications to understand how Pt_3_Te_4_ terminations tend to evolve in an H_2_O_2_ environment. To this end, density functional theory (DFT) simulations were performed to model the interaction between H_2_O_2_ molecules and the PtTe_2_ and Pt_2_Te_2_ terminations of Pt_3_Te_4_.

Theoretical simulations provide a clear rationale for the observed differences in the oxidation susceptibility between both terminations of Pt_3_Te_4_. To understand this behavior, we calculated the adsorption energy of H_2_O_2_ at both terminations. The adsorption of H_2_O_2_ on both PtTe_2_ (Figure 4a) and Pt_2_Te_2_ (Figure 4e) was energetically favorable, with adsorption energies of −8.9 and −20.3 kJ mol^–1^, respectively. After adsorption, H_2_O_2_ decomposition, which is an exothermic process on both terminations (−99.1 and –142.5 kJ mol^–1^ for PtTe_2_ and Pt_2_Te_2_, respectively), should be considered (Figure 4b,f, respectively). The subsequent step in the process is the migration of the second hydroxyl group to the oxidized Te atoms. This step is mildly exothermic for the Pt_2_Te_2_ surface termination (Figure 4g, energy gain of −9.8 kJ mol^–1^) and corresponds to the moderate energy cost for PtTe_2_ surface termination (Figure 4c, +23.3 kJ mol^–1^). The final step of the process is the removal of the Te atom via the formation of Te­(OH)_2_ molecules (Figure 4d,h). This step is endothermic for both terminations, but the energy cost for the Pt_2_Te_2_ surface (Figure 4h) is more than twice as large as that for the PtTe_2_ (Figure 4d) termination (+260.1 vs +118.3 kJ mol^–1^). The endothermic Te removal (Figure 4d,h) was overcome by the subsequent exothermic formation of TeO_2_ and PtO*_x_* products (Table 1), making the overall degradation of the PtTe_2_-terminated layers thermodynamically favorable. Conversely, the Pt_2_Te_2_-terminated surface underwent favorable oxidation, and the structures depicted in Figure 4f,g were similar in energy, leading to an equilibrium mixture. We then modeled the possible products of PtTe_2_ degradation with the formation of TeO_2_ and different oxides and hydroxides of platinum (see the reaction paths in Table 1). Considering the layered structure of PtTe_2_, a simulation of monolayer PtO_2_ was performed. Calculations suggest that bulk PtO_2_ is the most stable oxidation product (formation energy of −192.3 kJ mol^–1^), but the formation of a monolayer PtO_2_ in H_2_O_2_-modified Pt_3_Te_4_ is also likely, due to the similar magnitude of energy gain (−177.1 kJ mol^–1^), given the limited availability of Pt atoms, as previously observed on NO_2_-dosed Pt(111) in the temperature range of 500–700 K.^53^ In contrast, the formation of a Pt­(OH)_2_ layer corresponds to a smaller energy gain (−163.0 kJ mol^–1^); thus, it is less energetically favorable than bulk and monolayer PtO_2_. The energy gain for PtO formation (−182.8 kJ mol^–1^) was close to that for PtO_2_ formation. Thus, in principle, under-oxidized platinum could also be observed among the products of PtTe_2_ degradation, whereas platinum hydroxide is the least probable product.

**Figure 4:**
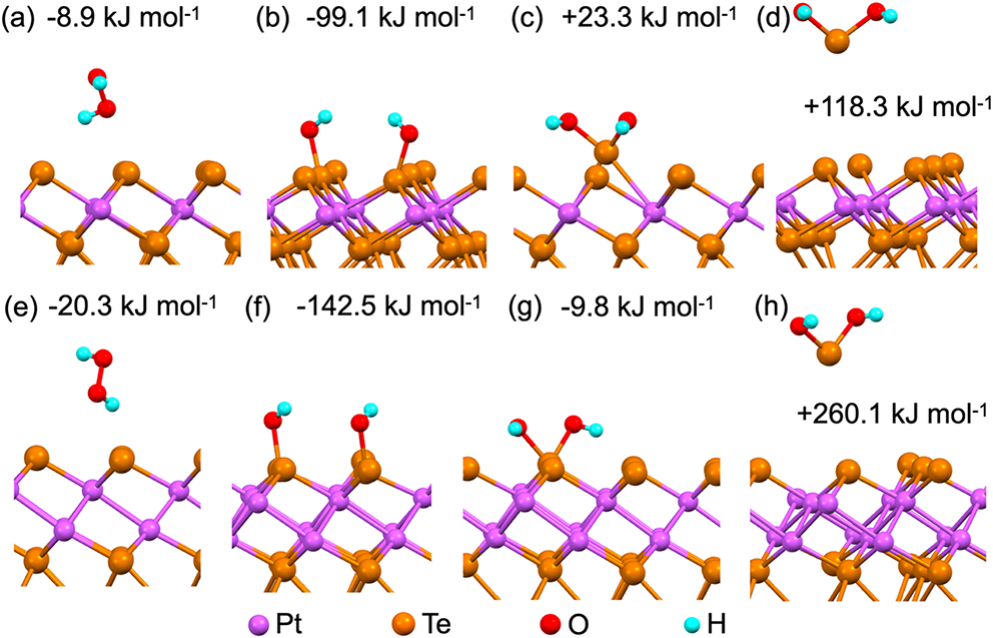
Optimized atomic structures and corresponding energies for (a–d) PtTe_2_ and (e–h) Pt_2_Te_2_ surface degradation by interaction with H_2_O_2_.

**Table 1:** Balanced Reactions for the Oxidative Decomposition of PtTe_2_ in the Presence of H_2_O_2_ Show the Possible Formation of PtO, Monolayer (2D) PtO_2_, or Pt­(OH)_2_ as Platinum-Containing Products, Together with TeO_2_ ^a^

reactants	products	reaction energy (kJ mol^–1^ H_2_O_2_^–1^)
${\mathrm{PtTe}}_{2}+5H_{2}O_{2}$	$\mathrm{PtO}+2{\mathrm{TeO}}_{2}+5H_{2}O$	–182.8
${\mathrm{PtTe}}_{2}+6H_{2}O_{2}$	${\mathrm{PtO}}_{2}\left(2D\right)+2{\mathrm{TeO}}_{2}+6H_{2}O$	–192.3 (−177.1)
${\mathrm{PtTe}}_{2}+5H_{2}O_{2}$	$\mathrm{Pt}{\left(\mathrm{OH}\right)}_{2}+2{\mathrm{TeO}}_{2}+4H_{2}O$	–163.0

a The reaction energies were reported per mole of H_2_O_2_. The value in parentheses refers to the energy associated with the formation of the PtO_2_ monolayer.

Simulations of the HER in acidic media were performed to assess the effect of oxidation on the catalytic properties of Pt_3_Te_4_ (Figure 5a), following methods reported elsewhere^54^ to model the impact of vacancies on the catalytic performance. The calculated free energy diagram demonstrates that the magnitude of the free energy for the rate-determining step of the reaction (hydrogen adsorption) is almost 1 eV smaller than the rate-determining step over the PtTe_2_ termination with Te vacancy (PtTe_1.80_) or Pt surface (−1.05 vs ∼ −0.09 eV). This step is endothermic for the defect-free PtTe_2_ termination of Pt_3_Te_4_. In our previous study on the catalytic properties of nonoxidized Pt_3_Te_4_, the improvement in efficiency was associated with changes in the electronic structure at the Fermi level.^54^ The oxidation of the surface corresponds to a substantial increase in the density of states at the Fermi level (Figure 5b). High catalytic performance also requires charge transfer from the bulk to the surface layer. The visualized redistribution after the formation of the PtO_2_/Pt_3_Te_4_ interface demonstrated significant charge density redistribution, which was associated with efficient charge transfer between the surface layer and bulk area (Figure 5c). Thus, based on these results, we propose that the oxidation of the PtTe_2_ termination can lead to a significant improvement in the electrochemical performance.

**Figure 5:**
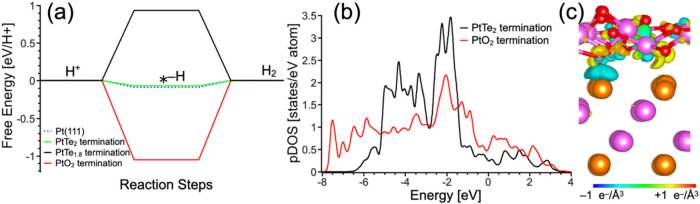
(a) Free energy diagram for the HER in acidic media over PtTe_2_ and PtO_2_ terminations. * indicates the substrate. (b) Partial density of states (pDOS) of the 5d orbitals of the Pt atoms in the PtTe_2_ and PtO_2_ terminations. Note that the “PtO_2_ termination” corresponds to a monolayer PtO_2_ on Pt_3_Te**_4_**; bulk PtO_2_ is not considered in the free-energy diagram/pDOS plot. (c) Change in the charge density after the formation of the PtO_2_/Pt_3_Te_4_ interface. The isosurface level is 0.01 e^–^/Å^3^.

### 2.3 Electrochemical Characterization

After establishing a theoretical framework for predicting the enhanced catalytic activity through the selective oxidation of PtTe_2_ termination, we experimentally validated these predictions by evaluating the electrochemical performance of Pt_3_Te_4_ nanosheets exfoliated with H_2_O_2_ (see the electrochemical parameters in Table 2). Linear sweep voltammetry in 0.5 M H_2_SO_4_ showed significant improvement in HER activity for oxidatively treated samples, with 0.3% H_2_O_2_-modified nanosheets showing a lower onset potential and overpotential at 10 mA cm^–2^ (Figure 6a and Table 2). This enhanced performance stems from the nanoscale porosity and partial oxidation induced by the H_2_O_2_ treatment. Moreover, the increased density of the exposed active sites resulting from surface nanostructuring contributed to the observed increase in the current density. A correlation was identified between oxidative treatment and catalytic performance, wherein higher concentrations of H_2_O_2_ resulted in reduced overpotentials and increased current densities. Specifically, the nanosheets treated with 0.3% H_2_O_2_ exhibited an onset potential of −25.9 mV and an overpotential of 78.7 mV at 10 mA cm^–2^, surpassing the performance of pristine Pt_3_Te_4_ nanosheets, which displayed values of 50.2 and 113.1 mV, respectively. Furthermore, the exchange current density increased from 0.106 to 0.347 mA cm^–2^. Tafel slope analysis showed that the H_2_O_2_-treated samples maintained slopes of 51–55 mV dec^–1^ (Figure 6b), suggesting an unchanged reaction pathway with improved kinetics owing to the increased number of active sites. Electrochemical impedance spectroscopy (EIS) confirmed the enhanced charge transfer properties, showing a reduced resistance at the electrode–electrolyte interface (Section S2, Supporting Information).

**Figure 6:**
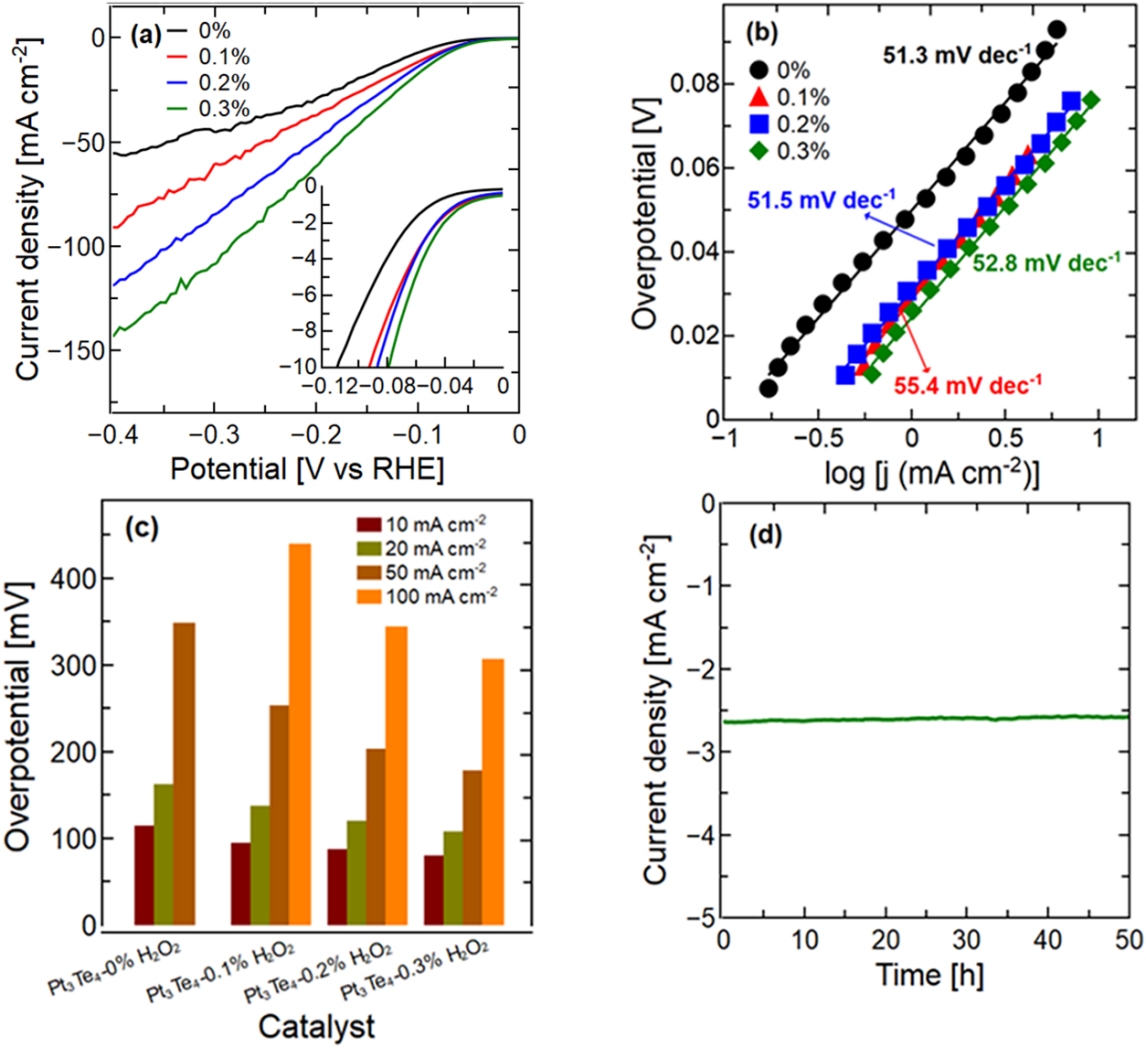
(a) Linear sweep voltammetry (LSV) measurements for Pt_3_Te_4_ samples, including the pristine nanosheets and those subjected to 0.1%, 0.2%, and 0.3% H_2_O_2_-assisted sonication. (b) The corresponding Tafel slopes for each sample were determined. (c) The overpotentials required to achieve current densities of 10, 20, 50, and 100 mA cm^–2^ were evaluated. (d) A chronoamperometric curve was recorded for the modified Pt_3_Te_4_ nanosheets (0.3% H_2_O_2_) in 0.5 M H_2_SO_4_ at a potential of −0.043 V (vs RHE).

**Table 2:** Electrochemical Parameters of the Tested Pt_3_Te_4_ Samples for HER in 0.5 M H_2_SO_4_

Pt_3_Te_4_ nanosheets prepared by LPE	*E*_onset_, mV at −1 mA cm^–2^	η, mV at −10 mA cm^–2^	*j*_0_, mA cm^–2^	Tafel slope, mV dec^–1^
0% H_2_O_2_	–50.2	113.1	0.106	51 ± 5
0.1% H_2_O_2_	–30.3	93.3	0.304	55 ± 5
0.2% H_2_O_2_	–33.1	86.0	0.253	52 ± 5
0.3% H_2_O_2_	–25.9	78.7	0.347	53 ± 5

Chronoamperometric tests at −0.043 V vs the reversible hydrogen electrode (RHE) demonstrated a stable current density over 50 h (Figure 6d). These results confirm that the oxidative exfoliation strategy enhances the catalytic performance while preserving the long-term stability of HER applications.

A comparison of the HER performances of the investigated catalysts with those of previously reported Pt-based and transition-metal dichalcogenides (TMDC)-based catalysts is shown in Table S3 in the Supporting Information. Definitely, nanoporous Pt_3_Te_4_ nanosheets prepared by H_2_O_2_-assisted LPE lower η_10_ from 113.1 to 78.7 mV and increase the exchange current density from 0.106 to 0.347 mA cm^–2^ with respect to pristine Pt_3_Te_4_, while keeping a Tafel slope of ∼53 mV dec^–1^. The same comparison in Table S3 highlights that this performance surpasses all previously reported single-phase Pt_3_Te_4_ and PtTe_2_ films and clearly outperforms prototypical TMDC catalysts such as VS_2_, WS_2_, and MoS_2_ nanotubes, approaching the activity of more complex Pt-based nanostructures and Pt/C benchmarks, yet obtained through a single scalable H_2_O_2_-assisted exfoliation step without additional dopants, supports, or secondary metallic phases.

Compared with previous results on bulk Pt_3_Te_4_ powders deposited on inert supports,^55^ the nanoporous flakes obtained in the presence of low H_2_O_2_ concentrations display a substantial decrease of the overpotential at 10 mA cm^–2^ and a marked increase of the exchange current density, while preserving a relatively low Tafel slope and long-term stability. In contrast to previous reports where Pt_3_Te_4_ is grown electrochemically on MoTe_2_ single crystals and benefits from a specific template and composite electrode architecture,^21,56^ here the catalytic response arises from free-standing Pt_3_Te_4_ nanosheets that can be processed as inks, which is more relevant for practical electrode manufacturing.

### 2.4 Operando AP-XPS Analysis

Harsh working environments, such as acidic media and applied potentials, limit the applicability of traditional XPS in operando studies. To overcome this limitation, we employed a dedicated electrochemical half-cell setup compatible with AP-XPS measurements.^57^ This configuration enabled us to monitor significant surface changes during the stepwise decrease in the applied voltage across different samples to reveal the underlying processes that govern the catalytic behavior during the HER. To capture the real-time evolution of the Pt-4f and Te-3d core levels, we varied the applied potential from 0 to −0.6 V. By comparing pristine Pt_3_Te_4_ nanosheets with those modified using 0.1% and 0.2% H_2_O_2_, we investigated the influence of surface oxidation on hydrogen adsorption, active site formation, and catalyst stability under operational conditions.

The operando AP-XPS spectra of the Pt-4f core level are shown in Figure 7. The spectrum recorded for the pristine sample (Figure 7a) is composed of two main components, Pt_2_Te_2_ and PtTe_2_, corresponding to the different surface terminations, emerging at binding energies of ∼71.5 and ∼72.4 eV,^20^ respectively. No significant changes were observed in these features up to −0.5 V bias voltage, meaning a consistent surface without major oxidation at lower cathodic potentials. However, at −0.6 V, we observed the emergence of a new peak at ∼73.5 eV, indicative of PtO_2_ formation^58,59^ on the PtTe_2_-terminated surfaces of Pt_3_Te_4_, which are more prone to oxidation (see Figure 4 and its discussion in Section 2.2). Notably, surface oxidation was significantly more evident in the Pt_3_Te_4_ nanosheets treated with 0.1% H_2_O_2_ (Figure 7b). While at 0 V the spectrum shows the same Pt_2_Te_2_ and PtTe_2_ components as in the pristine sample, we observed the PtO_2_ spectral feature in the Pt-4f core level already at −0.2 V. The intensity of the PtO_2_ peak increased with increasing absolute potential. This behavior implies that H_2_O_2_ treatment preconditions the surface and makes it more susceptible to the operating conditions, resulting in the formation of reactive oxygen species. It is plausible that during LPE, the addition of H_2_O_2_ increased the number of reactive Pt sites and surface defects, which eventually facilitated the formation of oxides. The most pronounced effect was observed in the sample treated with 0.2% H_2_O_2_ (Figure 7c), where the PtO_2_ component became detectable at the Pt-4f core level, even at 0 V, and dominated the spectra at different potentials with a clear tendency. This indicates that a higher H_2_O_2_ concentration during exfoliation significantly increased the oxidative reactivity of the surface. The presence of 2D PtO_2_ corresponds to the improved HER performance observed in the electrochemical measurements (Table 2 and Figure 6a) because PtO_2_ promotes hydrogen adsorption and serves as the active catalytic phase.^60^ The functionality of oxidized Pt species could be a key factor in achieving high catalytic efficiency while maintaining surface stability. Beyond the BE of 76 eV, we observed additional spectral features whose origins were connected to the PtO_6_ and platinum hydroxide groups, possibly involving sulfur species. This was likely caused by interactions between the acidic H_2_SO_4_ electrolyte and the membrane components.

**Figure 7:**
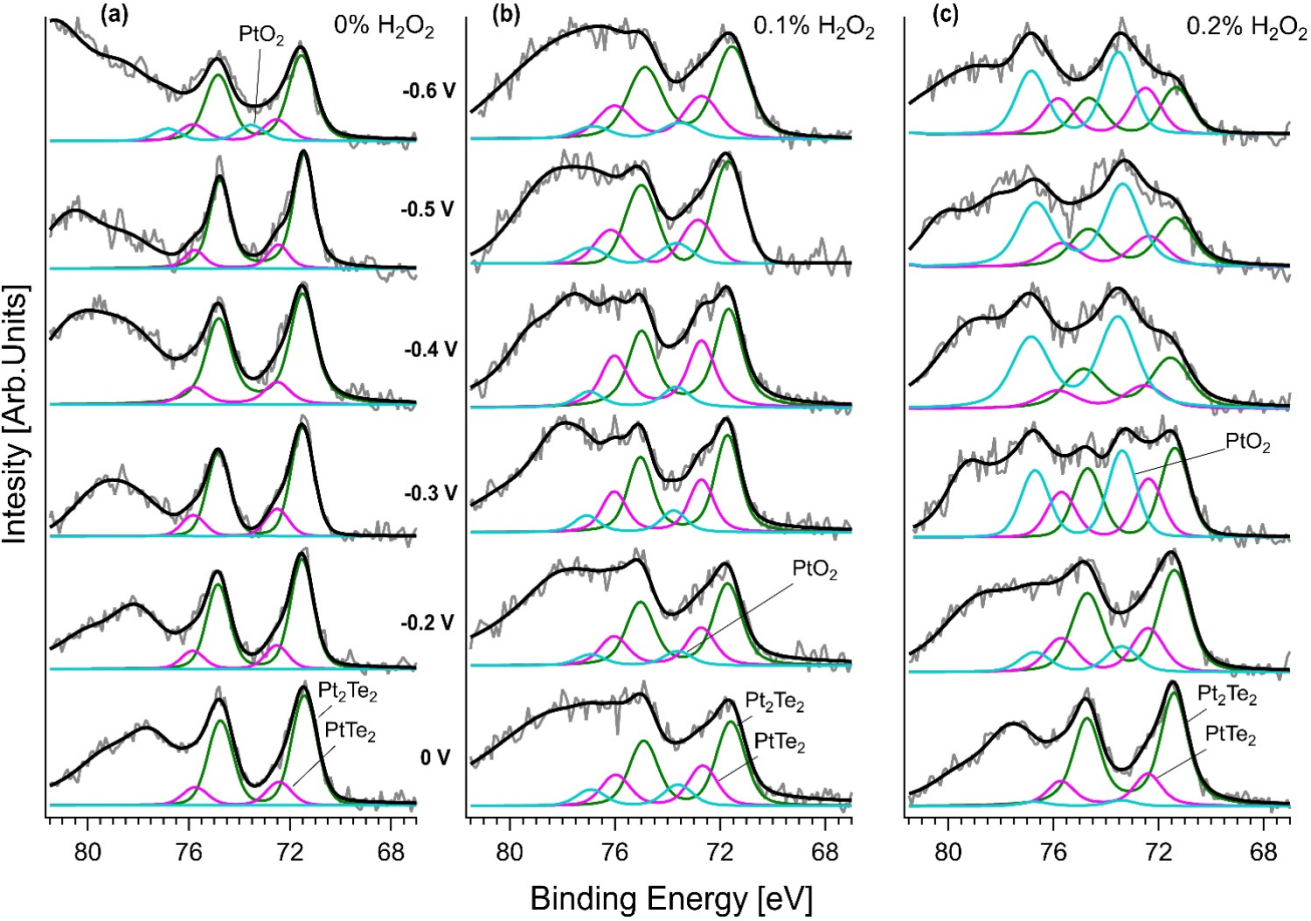
Operando AP-XPS analysis of the Pt-4f core level acquired with the EnviroESCA system during electrochemical measurements in a working half-cell. The spectra are shown as a function of the applied potential, stepped from 0 to −0.6 V, for three differently exfoliated samples of Pt_3_Te_4_ nanosheets, obtained by LPE in IPA (a) in the absence and with (b) 0.1% and (c) 0.2% H_2_O_2_.

Distinct changes were observed across different conditions and samples in the Te-3d core-level spectra (Figure 8). The spectrum of pristine Pt_3_Te_4_ nanosheets (Figure 8a) showed a dominant peak at ∼572.8 eV,^20,54,55^ associated with Te in the Pt_3_Te_4_ lattice, and a minor component at ∼575.0 eV, attributed to TeO_2_.^21^ The intensity of the TeO_2_ peak increased with more negative potentials (particularly beyond −0.4 V), suggesting that Te oxidation is potential-dependent and occurs as the HER proceeds. However, this oxidation was relatively limited in the pristine sample, consistent with the stability of Pt-4f under the same conditions. This situation changed for H_2_O_2_-modified Pt_3_Te_4_ nanosheets. In the 0.1% H_2_O_2_-treated sample (Figure 8b), the TeO_2_ peak was more pronounced and appeared at lower potentials than in the pristine case. This early oxidation indicates that H_2_O_2_-assisted LPE not only affects the Pt sites but also alters the local chemical environment of Te, making it more prone to oxide formation during electrolysis. The 0.2% H_2_O_2_-treated sample (Figure 8c) exhibited a dominant TeO_2_ peak across the entire potential range, further highlighting the role of surface modification.

**Figure 8:**
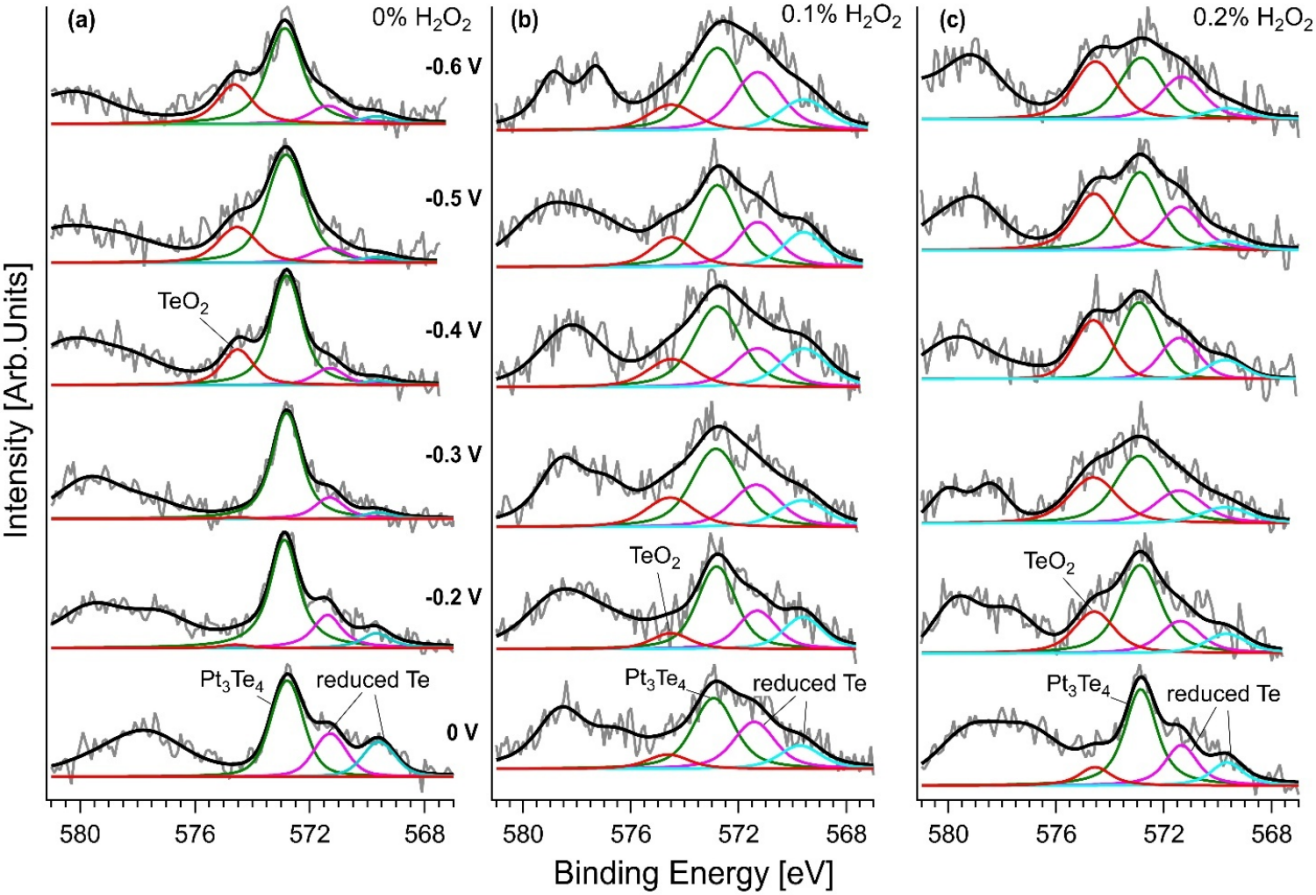
Operando AP-XPS analysis of the Te-3d_5/2_ core level was performed using an EnviroESCA system during electrochemical measurements in a working half-cell. The spectra are shown as a function of the applied potential, stepped from 0 to −0.6 V, for three differently exfoliated samples of Pt_3_Te_4_ nanosheets, obtained by LPE in IPA (a) in the absence and, moreover, with (b) 0.1% and (c) 0.2% H_2_O_2_.

The sample modified with a higher (0.2%) concentration of H_2_O_2_ underwent more extensive oxidation, which was also characterized by stable oxidized states under cathodic bias. Additionally, the emergence of reduced components at ∼571.4 and ∼569.6 eV highlights the coexistence of oxidation and reduction processes, suggesting that the hydrogenation of Te occurs concurrently with oxidation. This reduction is likely due to the interaction of hydrogen atoms with undercoordinated sites, forming Te–H bonds, which results in a lower BE. The simultaneous observation of reduced Te species suggests a complex interplay between oxidation and hydrogenation,^61–63^ which may enhance the catalytic behavior by dynamically tuning the surface reactivity.

It is important to note that driving the formation of platinum oxides by electrical polarization under HER-relevant conditions is intrinsically challenging. In the potential–pH window where the HER proceeds, the thermodynamic basin favors metallic Pt and rapidly reduces any Pt–O species; therefore, the applied cathodic bias cleans the surface of oxygenated adsorbates and accelerates the competitive pathways that remove oxides, including chemical reduction by adsorbed H and dissolution–redeposition cycles. Kinetically, proton discharge outcompetes oxygen insertion. The compact double layer screens the nucleation of O-containing species, and even when oxidants are present, their lifetime at a negatively polarized interface is short, making the sustained appearance of Pt­(II) and Pt­(IV) signatures unlikely to occur. Moreover, a thick insulating PtO_2_ film would quench charge transfer; therefore, any oxide that survives under load must remain ultrathin, conductive, and epitaxially stabilized at specific sites, an exacting constraint rarely met by Pt. Against this backdrop, observing a reversible, bias-activated PtO_2_ skin on PtTe_2_-terminated surfaces of Pt_3_Te_4_ is notable: hydrogen peroxide preconditioning seeds a small population of reactive terminations, whereas the interfacial field under cathodic polarization selectively stabilizes a subnanometric oxide at these terminations without passivating the electrode, revealing an unconventional, field-stabilized route to platinum oxidation that is normally disfavored during the HER. Correspondingly, the subnanometric thickness of the bias-activated 2D PtO skin on the PtTe_2_-terminated surfaces of Pt_3_Te_4_ was confirmed by quantitative XPS analysis using survey spectra under the same experimental conditions as those related to the spectra in Figures 7–8, following the methods in refs 64,65 Specifically, to estimate the thickness of the oxide skin, we converted the Pt 4f oxide/metal area ratio into an equivalent Pt-oxide thickness (see also Section S3 in the Supporting Information). For the 0.2% H_2_O_2_-modified sample, the thickness of the oxide skin increased from 0.06 ± 0.06 nm at 0 V up to 1.16 ± 0.23 nm at −0.5 V (with neglible modifications for −0.6 V), reflecting the emergence of a higher-BE PtO_2_ component under cathodic bias.

## 3 Conclusion

By coupling H_2_O_2_-assisted LPE with SPEM and operando in-cell AP-XPS, we show that the electrochemical bias drives the selective formation of PtO_2_ on the PtTe_2_-terminated regions of Pt_3_Te_4_ nanosheets, while the Pt_2_Te_2_ terminations remain metallic, constituting the catalytically active interface for the HER. Mild H_2_O_2_-assisted LPE preserves bulk crystallinity while introducing nanoscale porosity and rugged edge motifs, whose density scales with the oxidant concentration without detectable oxygen incorporation into the crystal interior, thus activating the basal plane beyond the edge sites.

Nanostructuring the basal plane of Pt_3_Te_4_ nanosheets by H_2_O_2_-assisted LPE translates into practical HER gains: the nanoporous nanosheets (0.3% H_2_O_2_) lower the overpotential at 10 mA cm^–2^ by ∼ 30% (from 113.1 to 78.7 mV), advance the onset potential by 24 mV (from −50.2 to −25.9 mV), and boost the exchange current density more than 3-fold (0.106 → 0.347 mA cm^–2^), while maintaining similar Tafel slopes (∼51–55 mV dec^–1^) and stable operation for 50 h in acid. Electrochemical impedance spectroscopy revealed exceptionally fast adsorption/desorption kinetics, consistent with accelerated charge transfer at the electrolyte interface.

Mechanistically, SPEM resolves the termination map and shows that H_2_O_2_ treatment converts PtTe_2_-like domains while preserving Pt_2_Te_2_ metallic regions. Operando AP-XPS then captured the earlier emergence of PtO_2_ under cathodic bias only on the treated nanosheets, which was already visible at −0.2 V (vs RHE) compared to ≈ −0.6 V of pristine, together pinpointing PtTe_2_-terminated regions as reactive loci. In parallel, Te remains largely stable with potential-dependent reversible signatures (partial oxidation/hydrogenation) at under-coordinated sites. Theoretical modeling demonstrated that H_2_O_2_ adsorbed and decomposed more favorably on PtTe_2_ than on Pt_2_Te_2_, lowering the energetic cost for oxide formation and yielding 2D PtO_2_ skin, which increased the density of states around the Fermi level and facilitated interfacial charge redistribution, which together reduced the free-energy barrier for the rate-determining HER step.

Lastly, this approach is scalable (LPE, low loadings, and mild oxidants) and avoids extrinsic dopants or plasma/ion beams, offering a cost-effective path to uniform defect landscapes.

## 4 Methods

### 4.1 Single-Crystal Growth

Single crystals of Pt_3_Te_4_ were grown by the self-flux method. Unlike the case of PtTe_2_,^66^ the growth window of Pt_3_Te_4_ is narrow. The mixtures of high-purity Pt foil and Te ingots with the molar ratio of 51:49 were inserted in an alumina crucible and sealed into an evacuated quartz ampule. The quartz ampule was heated to 1080 °C for 24 h and then slowly cooled to 975 °C at a rate of 1 °C/h. The excess flux was separated by centrifugation above 970 °C and mechanical polishing. Shiny platelike Pt_3_Te_4_ single crystals were harvested with a dimension of 4 × 3 × 0.4 mm^3^.

### 4.2 Liquid-Phase Exfoliation

Pt_3_Te_4_ nanosheets were exfoliated in the liquid phase in atomically thin layers in two configurations: (i) pristine nanosheets and (ii) modified nanosheets prepared via H_2_O_2_-assisted exfoliation. The pristine configuration involved the fine-graining of bulk Pt_3_Te_4_ crystals and their dispersion in isopropanol (IPA) at a concentration of 1 mg/mL. The same exfoliation protocol was followed for the modified nanosheets, but hydrogen peroxide (H_2_O_2_) was added to the IPA solvent before sonication at concentrations of 0.1%, 0.2%, and 0.3%.

### 4.3 HER

The HER on Pt_3_Te_4_ nanosheets was investigated by linear sweep voltammetry (LSV) in a 0.5 M H_2_SO_4_ solution using a PGSTAT302 potentiostat with a three-electrode configuration. The working electrode was glassy carbon (GC) coated with Pt_3_Te_4_ nanosheets obtained via LPE: pristine nanosheets without additives and modified nanosheets obtained via H_2_O_2_-assisted LPE. Pt and Ag/AgCl were used as the counter and reference electrodes, respectively. LSVs were recorded in N_2_-saturated 0.5 M H_2_SO_4_ at 2 mV s^–1^ and 1600 rpm, with the potentials referenced to RHE. The current densities were normalized to the geometric area of the catalysts. The GC electrode (0.196 cm^2^) was modified with 12 μL of Pt_3_Te_4_ ink at a loading of 186.7 μg cm^–2^. The samples were stabilized by cyclic voltammograms in 0.5 M H_2_SO_4_ at 50 mV s^–1^. Chronoamperometric testing was conducted at −0.043 V for 50 h. Electrochemical impedance spectroscopy was performed on a Zahner Zennium workstation in deaerated 0.5 M H_2_SO_4_ using a 10 mV amplitude from 10 kHz to 10 mHz.

### 4.4 SEM

SEM analysis was conducted using GeminiSEM 500, a field-emission SEM equipped with a Schottky-type hot cathode operating in a voltage range of 0.02–30 kV. The operational mode was set at 3 kV, utilizing both the in-lens and backscattering acquisition modes.

### 4.5 AFM

AFM images were acquired using a Digital D5000 Veeco system operating in tapping mode with P-doped *n*-type Si cantilevers from Veeco.

### 4.6 TEM

The samples for TEM were prepared by depositing a droplet of a liquid suspension containing Pt_3_Te_4_ flakes onto a Cu grid with a holey carbon membrane. TEM investigations were performed using a JEM ARM200F analytical TEM instrument operated at 200 kV.

### 4.7 SPEM

SPEM measurements were conducted using a scanning photoelectron microscope at the Elettra Esca Microscopy beamline. In this configuration, a Fresnel zone plate optics system focuses the X-ray beam onto a spot with a diameter of approximately 120 nm. By scanning the sample relative to the focused X-ray beam, we created chemical and topographical maps, while simultaneously collecting element-specific photoelectrons.

### 4.8 AP-XPS

An Electron Spectroscopy for Chemical Analysis under Environmental Conditions (EnviroESCA) apparatus developed by SPECS GmbH (Germany) was used to study the surface chemistry of Pt_3_Te_4_ nanosheets using AP-XPS. The system used a monochromatic Al Kα X-ray source (photon energy of 1486.6 eV) and a Phoibos 160 NAP hemispherical analyzer with a one-dimensional delay line detector, operated at a pass energy of 20 eV. Dispersions of exfoliated Pt_3_Te_4_ nanosheets were spray-coated onto Nafion 212 membranes using a SonoTek ExactaCoat system, with a catalyst loading of 0.1875 mg cm^–2^. Membranes (2 × 2 cm^2^) with an active area of 1 × 1 cm^2^ were prepared in duplicate for reproducibility. Operando AP-XPS spectra were acquired while exposing the membrane surface to an acidic electrolyte environment, enabling the direct monitoring of the Pt-4f and Te-3d core levels under bias to understand the surface transformations during catalysis.

### 4.9 Theory

The atomic structure and energetics of various configurations and interactions were studied using DFT with the QUANTUM-ESPRESSO code,^67^ and GGA–PBE^68^ and vdW-like force correction^69^ were also used. Ultrasoft pseudopotentials were used.^70^ The energy cutoff values were 35 and 400 Ry for the plane-wave expansion of the wave functions and charge density, respectively. The physisorption enthalpies were calculated using the following standard formula

$${\Delta H}_{\mathrm{phys}}=\left[E_{\mathrm{subst}+\mathrm{mol}}-\left(E_{\mathrm{subst}}+E_{\mathrm{mol}}\right)\right]$$

where *E*_subst_ is the total energy of the substrate, and *E*_mol_ is the energy of the single molecules of the selected species in an empty box. Only the gaseous phase was considered for water adsorption. Decomposition energy is defined as the difference between the oxygen molecules on the surface. For physisorption, we evaluated the differential Gibbs free energy at a given temperature using the following formula

ΔG=ΔH−TΔS

where *T* is the temperature and Δ*S* is the change in entropy of the adsorbed molecule, which was estimated by considering the gas-to-liquid transition using the following standard formula

$$\Delta S={\Delta H}_{\mathrm{vaporisation}}/T$$

where Δ*H*_vaporization_ is the measured enthalpy of vaporization.

To simulate the surface of the bulk Pt_3_Te_4_ crystal, we used a slab constructed from 3 × 3 × 3 supercell. To imitate the effect of the rigid subsurface of the bulk crystal, we performed calculations using the fixed lattice parameters obtained from the calculations for the bulk crystal. Only the atomic positions were optimized in this study.

## Supplementary Material



## Notes

Additional TEM and HRTEM characterization of pristine and H_2_O_2_-treated Pt_3_Te_4_ nanosheets, including SAED indexing and EDS compositional analysis; supplementary discussion of treatment-induced edge disorder and pore-like features; electrochemical impedance spectroscopy (EIS) spectra with equivalent-circuit fitting parameters; Pt-oxide equivalent thickness estimation from Pt 4f spectra using an overlayer model (equation S1) with tabulated thickness values versus applied potential; benchmarking table comparing HER metrics of Pt_3_Te_4_ with Pt-based catalysts and representative transition-metal dichalcogenides (PDF)
