# Supplementary material for: Scalable Defect Engineering of Pt3Te4 Nanosheets Activates an Electro-Switchable and Termination-Dependent PtO2 Skin for Low-Overpotential Hydrogen Evolution
Source: ACS Appl Mater Interfaces. 2026 Jan 27;18(5):8026–38. doi: 10.1021/acsami.5c18460 (PMC12903106; doi:10.1021/acsami.5c18460)
Supplement: Supplementary file 1 [file am5c18460_si_001.pdf]

Supporting Information

Scalable Defect Engineering of Pt_3Te_4 Nanosheets Activates an Electro-Switchable and Termination-Dependent PtO_2 Skin for Low-Overpotential Hydrogen Evolution

Tsotne Dadiani [1,§], Gianluca D'Olimpio[1], Loreta Tamašauskaitė-Tamašiūnaitė[2,§], Stefano Zenone[1,3], Chia-Nung Kuo[4,5,6], Matteo Amati[7], Zygmunt Milosz[7], Luca Gregoratti[7], Tomáš Hrbek[8], Miquel Gamón Rodríguez[8], Marian Cosmin Istrate[9], Chin Shan Lue[4,5,6], Yevheniia Lobko[8], Corneliu Ghica[9], Eugenijus Norkus[2], Yong-Wei Zhang[10], Anna Cupolillo[11,*], Danil W. Boukhvalov[12,13,*], Antonio Politano[1,*]

[1] *Department of Physical and Chemical Sciences, University of L'Aquila, via Vetoio, 67100 L'Aquila (AQ), Italy*
[2] *Department of Catalysis, Center for Physical Sciences and Technology, Saulėtekio Ave. 3, LT-10257, Vilnius, Lithuania*
[3] *Department of Applied Science and Technology, Polytechnic University of Turin, Corso Castelfidardo, 39, 10129 Turin, Italy*
[4] *Program on Key Materials, Academy of Innovative Semiconductor and Sustainable Manufacturing (AISSM), National Cheng Kung University, Tainan 70101, Taiwan*
[5] *Department of Physics, National Cheng Kung University, Tainan 70101, Taiwan*
[6] *Taiwan Consortium of Emergent Crystalline Materials (TCECM), National Science and Technology Council, Taipei 10601, Taiwan*
[7] *Elettra – Sincrotrone Trieste SCpA, AREA Science Park, Strada Statale 14 km 163.5, 34149, Trieste, Italy*
[8] *Dept. Surface and Plasma Science, Faculty of Mathematics and Physics, Charles University, V Holešovičkách 2, 180 00, Prague, Czech Republic*
[9] *National Institute of Materials Physics, Atomistilor 405A, 077125 Magurele, Romania*
[10] *Institute of High Performance Computing (IHPC), Agency for Science, Technology and Research (A*STAR), Singapore 138632, Republic of Singapore*
[11] *Department of Physics, University of Calabria, Via P. Bucci cubo 31/C, Rende, CS, 87036 Italy*
[12] *College of Science, Institute of Materials Physics and Chemistry, Nanjing Forestry University, Nanjing 210037, P. R. China*
[13] *Institute of Physics and Technology, Satbayev University, Ibragimov str. 11, Almaty, 050032 Kazakhstan*

§ These authors contributed equally

*corresponding authors:

Prof. Anna Cupolillo (anna.cupolillo@fis.unical.it); Prof. Danil Boukhvalov (danil@njfu.edu.cn); Prof. Antonio Politano (antonio.politano@univaq.it);

S.1 HRTEM measurements

S.1.1 Pristine Pt_3Te_4 nanosheets (LPE with 0.0% H_2O_2)

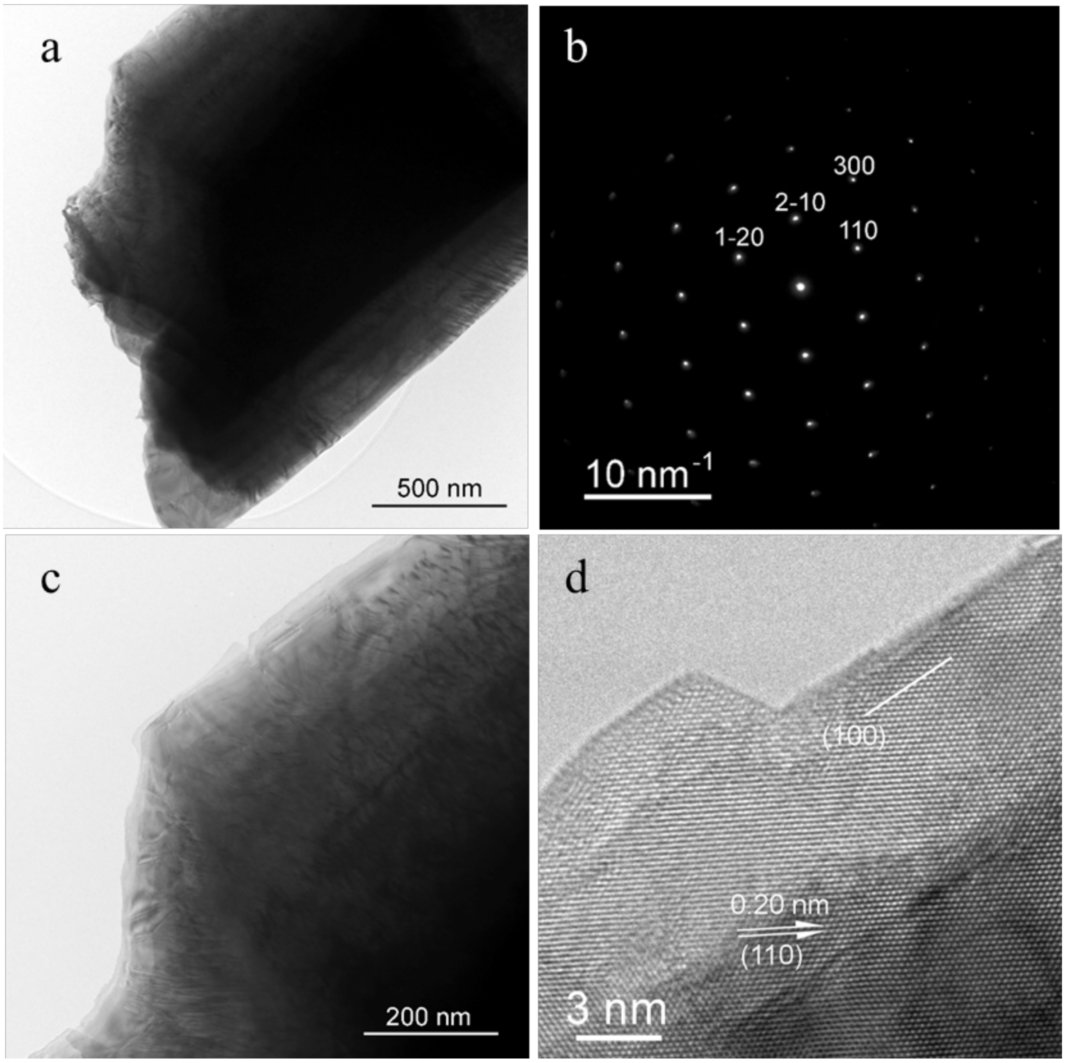

Figure S1. (a) Typical micrometric Pt_3Te_4 flake from sample Pt3Te4-0.0% and (b) the associated SAED pattern along the [001] zone axis; (c) and (d) high-magnification TEM and HRTEM images in the [001] orientation showing a step-like thickness near the flake border due to exfoliation on the (001) planes.

The TEM image in Fig. S1a exhibits a typical micrometric flake, along with the associated SAED pattern (Fig. S1b). The associated SAED pattern acquired from a large area on this flake was indexed according to the trigonal structure of Pt_3Te_4, space group R-3m (no. 166), having as lattice parameters a=b=0.3988 nm, c=3.5390 nm, α=β=90º, γ=120º (cif no. 8104024). Pattern indexation corresponded to the **B**=[001] zone axis.

The layered nature of the Pt_3Te_4 crystal structure is revealed by the step-like graded contrast close to the flake border, which is noticeable at higher magnifications or in the HRTEM micrographs. The HRTEM image in Fig. S1d shows that the thickness steps and border of the grain are defined by 100 crystallographic planes.

The chemical elemental composition of the sample was analyzed by energy-dispersive X-ray spectroscopy (EDS), and the spectrum acquired from this grain is presented in Fig. S2. The major features are the main peaks of Pt and Te in the sample, while the other peaks (Cu, C) were generated by electron scattering onto the TEM copper grid and carbon membrane. The quantitative analysis of the EDS spectrum relative to the elements of interest provides an atomic ratio Pt/Te = 0.76, which is very close to the 0.75 nominal atomic composition of Pt_3Te_4, and is reproducible in all the spectra acquired from different locations of the sample.

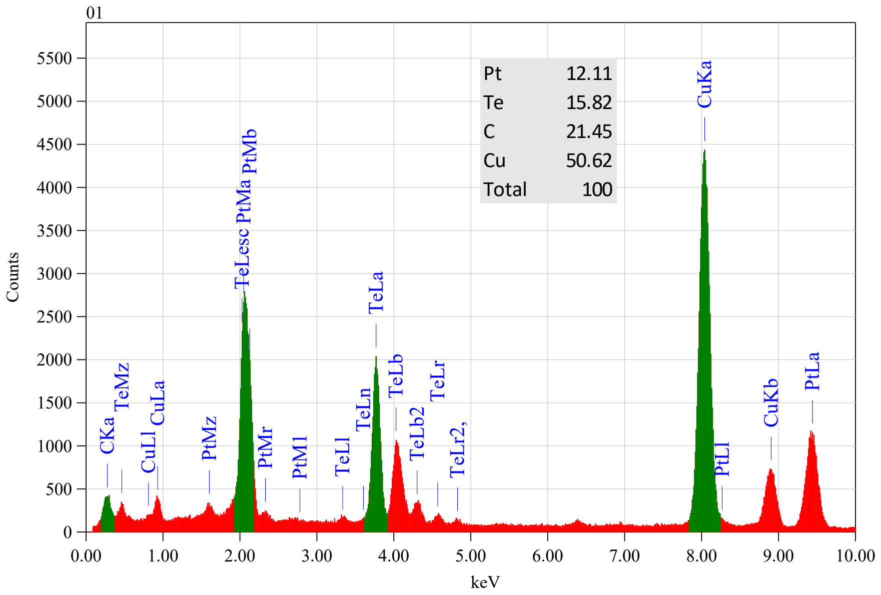

Fig. S2. EDS spectrum of the Pt_3Te_4 flake in figure 3; atomic ratio Pt/Te=0.76.

S.1.2. Pt_3Te_4 nanosheets (LPE with 0.1% H_2O_2)

TEM observations revealed micrometric flake-like grains in this case (Fig. S3). The associated SAED pattern (Fig. S3b) acquired from a large area on the right side of this flake was indexed according to the trigonal structure of Pt_3Te_4, space group R-3m (no. 166), having as lattice parameters

a=b=0.3988 nm, c=3.5390 nm, α=β=90°, γ=120° (cif no. 8104024). Pattern indexation corresponded to the **B**=[001] zone axis.

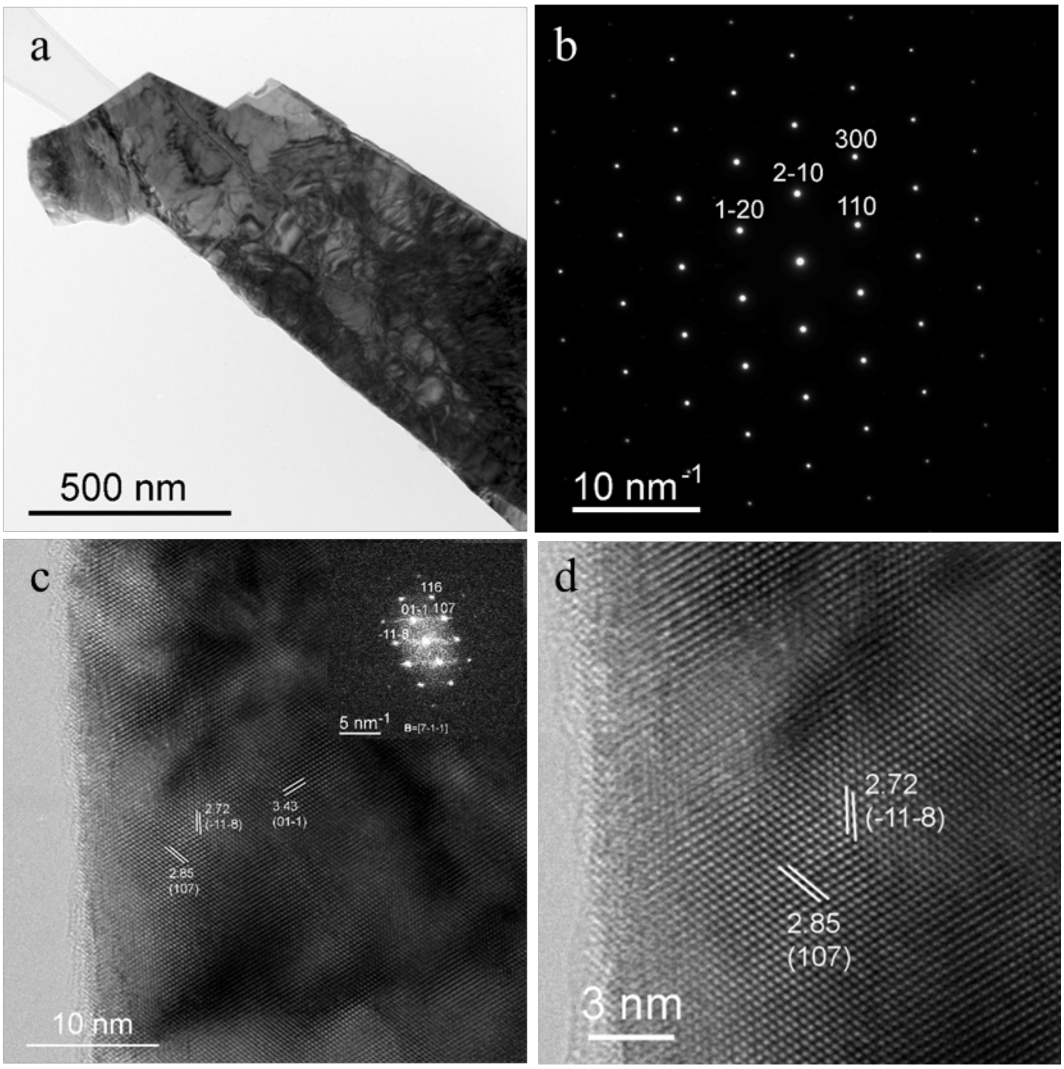

Figure S3. (a) Fragment from a Pt_3Te_4 flake produced by LPE with 0.1% H_2O_2 and (b) associated SAED pattern along the [001] zone axis from the central part of the flake; (c) and (d) HRTEM images close to the grain border in a thin area (upper left side) in the [7-1-1] orientation.

The high-resolution TEM (HRTEM) images in Fig. S3c and d were acquired from the thinner area on the upper-left side of the flake, with a slightly different crystallographic orientation, **B**=[7-1-1], with respect to the main part of the flake.

The chemical elemental composition of the sample was analyzed by EDS, and the spectra acquired from this grain are presented in Fig. S3. The major features are the main peaks of Pt and Te in the sample, while the other peaks (Cu, C) were generated by electron scattering onto the TEM copper grid and carbon membrane. The quantitative analysis of the EDS spectrum relative to the elements of interest, provides the atomic ratio Pt/Te = 0.79, very close to the 0.75 nominal atomic composition of Pt_3Te_4, reproducible in all the spectr acquired from different locations of thewhich is sample.

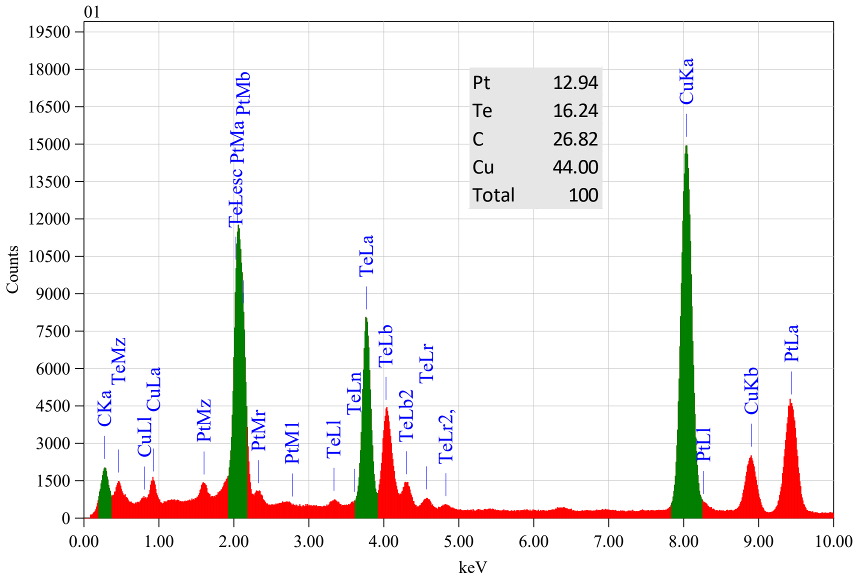

Figure S4. The EDS spectrum of the Pt_3Te_4 flake in Fig.and is S3; atomic ratio Pt/Te=0.79.

S.1.2 Pt_3Te_4 nanosheets (LPE with 0.2% H_2O_2)

A TEM sample typically consists of micrometric flakes made up of large-area structural domains measuring hundreds of nanometers in lateral size. The TEM image of a typical flake is shown in Fig. S5 along with the associated SAED patterns. The layered architecture of the Pt_3Te_4 crystal structure was suggested by the step-like graded contrast close to the flake border. The associated SAED pattern acquired from a large area of this flake reveals a single-crystal pattern showing a 6-fold symmetry. The diffraction pattern has been indexed according to the trigonal structure of Pt_3Te_4, space group R-3m (no. 166), having as lattice parameters a=b=0.3988 nm, c=3.5390 nm, α=β=90°, γ=120° (cif no. 8104024). Pattern indexation corresponded to the **B**=[001] zone axis. Rounded features of ~ 3 nm showing a pore-like contrast may be observed along the thin border of the grain (Fig. S5c,d). Considering the reduced material thickness along the edge of this flake, we have reasons to consider that the observed features are not actually fully embedded pores, but rather surface cavities or indentations, possibly one- or two-unit cells deep (3.5 – 7 nm measured along the c axis), as a result of the H_2O_2 treatment.

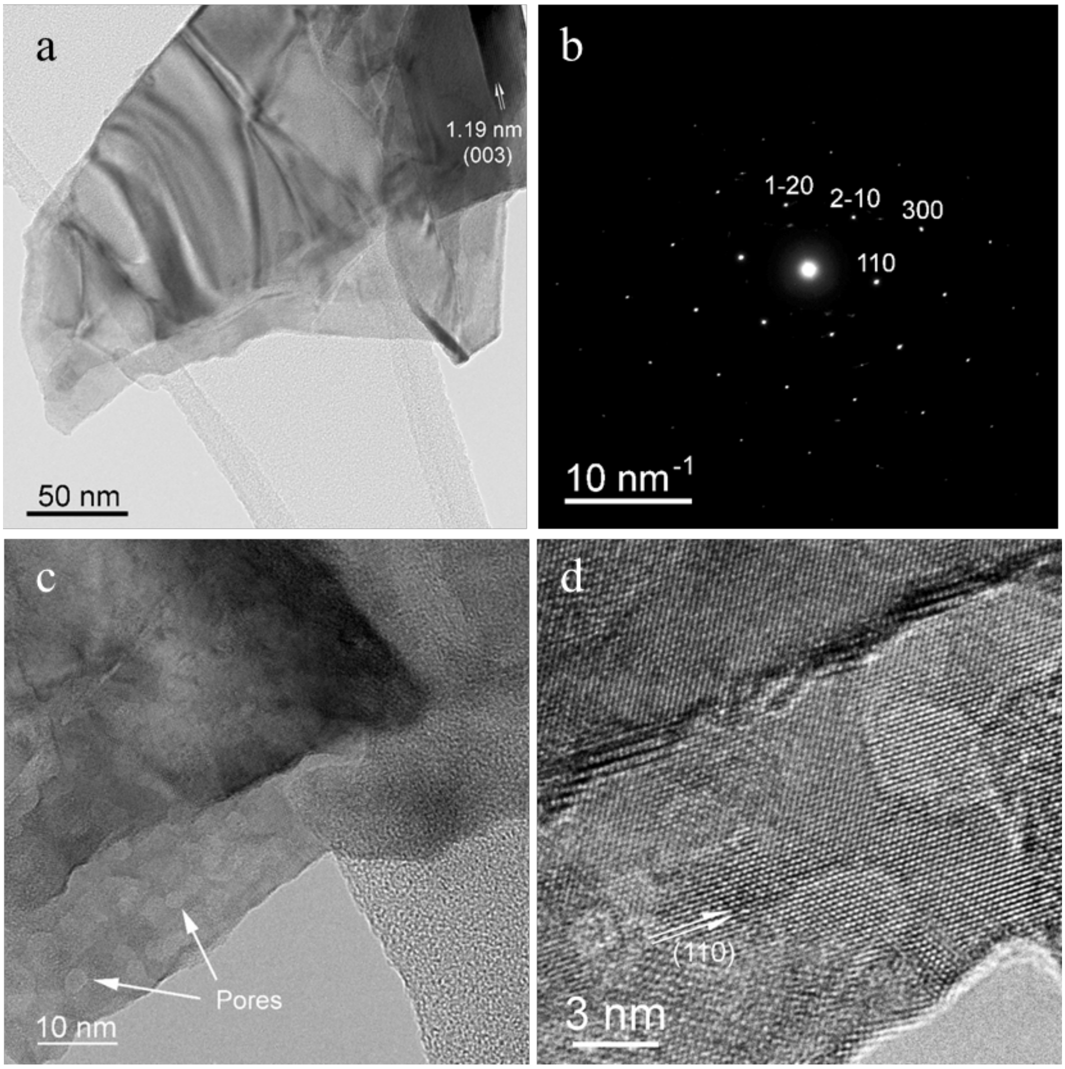

Figure S5. (a) Fragment from a typical Pt_3Te_4 flake produced by LPE with 0.2% H_2O_2; (b) Associated SAED pattern of the flake, close to the [001] zone axis orientation; (c) and (d) HRTEM images in the [001] orientation showing a step-like thickness near the flake border due to exfoliation on (001) planes.

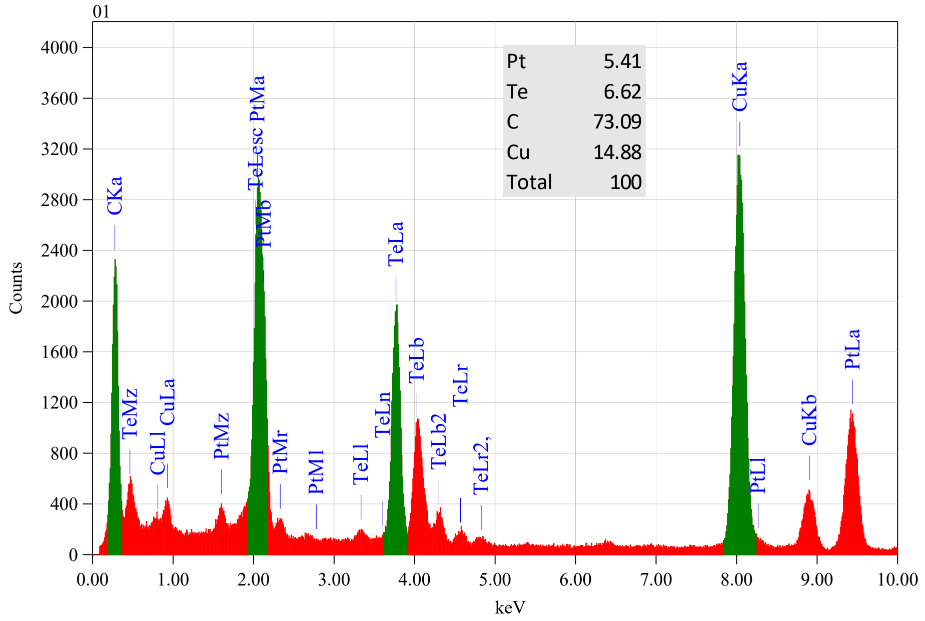

Figure S6. The EDS spectrum of the Pt_3Te_4 flakes shown in Fig. S5; Pt/Te atomic ratio =0.81.

The chemical elemental composition of the sample was analyzed by EDS, and the spectra acquired from this grain are presented in Fig. S6. The major features are the main peaks of Pt and Te in the sample, while the other peaks (Cu, C) were generated by electron scattering onto the TEM copper grid and carbon membrane. The quantitative analysis of the EDS spectrum relative to the elements of interest, provides the atomic ratio Pt/Te = 0.81, very close to the 0.75 nominal atomic composition of Pt_3Te_4, reproducible in all the spectra acquired from different locations of the sample.

By comparing the TEM images acquired for the pristine and the H_2O_2 treated samples Pt_3Te_4 samples, a few morphological and structural features can be noticed that can be connected to the applied chemical treatment. The HRTEM micrograph of the pristine sample (figure S1d) shows perfectly crystallized grain edges parallel to the {100} crystallographic planes of the trigonal Pt_3Te_4 lattice.

In the case of Pt_3Te_4 prepared by LPE with 0.1% H_2O_2, a thin (2-5 nm) disordered or amorphous layer may be observed along the thin edges of the grains. Two images are shown in Fig. S7. The band with black and white stripes parallel to the grain edge is a pyramidal planar defect formed during the Pt_3Te_4 growth process. Such disordered edges could not be observed in the case of the pristine sample, where the HRTEM images exhibited a well-crystallized status to the very limit of the grains. Therefore, we consider that the formation of structurally disordered surface layers in the case of the Pt_3Te_4 nanosheets obtained by LPE with 0.1% H_2O_2 is the consequence of chemical treatment in H_2O_2.

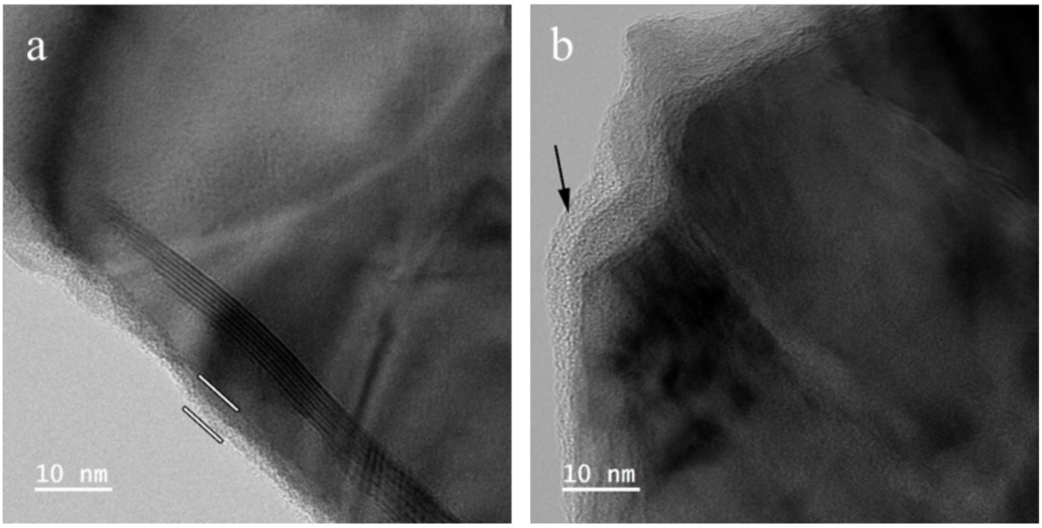

Figure S7. (a) High-resolution TEM image of a Pt_3Te_4 nanosheet produced by LPE with 0.1 % H_2O_2, showing the crystalline interior and the edge region. (b) Magnified view of the same edge region, highlighting the disordered/amorphous layer at the outermost part of the crystal grain (arrow).

.

In the case of the Pt3Te4-0.2% sample, the formation of pore-like features and rugged edges, as observed in the thinnest areas of the analyzed flakes, may also be related to the chemical treatment applied in H_2O_2 (figure 8).

The effects of the oxidative chemical treatment seem to increase in intensity with the H_2O_2 concentration, from surface disorder in the case of the Pt_3Te_4-0.1% H_2O_2 sample to the formation of surface pores and rugged edges for the Pt_3Te_4-0.2% H_2O_2 sample. In none of the three samples could be confirmed by EDS, within the sensitivity limits of our instruments, the presence of oxygen in the analyzed Pt_3Te_4 grains as a result of the chemical oxidative treatment.

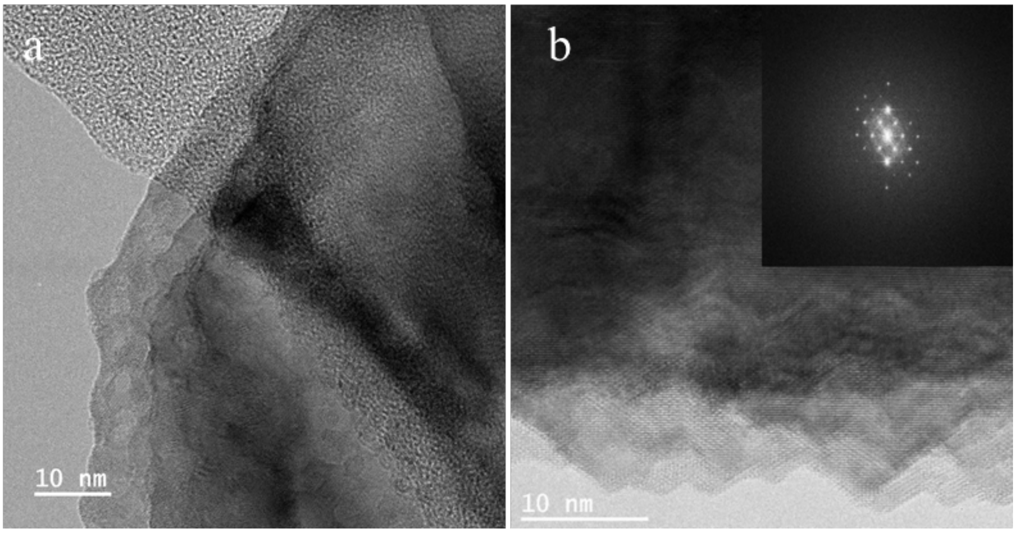

Figure S8. a) High-resolution TEM image of a Pt_3Te_4 nanosheet produced by LPE with 0.2 % H_2O_2, recorded in a very thin region close to the flake edge, where pore-like features and rugged edges develop as a result of the oxidative treatment. (b) High-resolution TEM image taken on a thicker region of the same flake, showing well-resolved lattice fringes; the inset reports the corresponding selected-area electron diffraction pattern, confirming the crystalline nature of Pt_3Te_4 after H_2O_2 treatment.

S2. EIS Measurements

Electrochemical impedance spectroscopy (EIS) was carried out in 0.5 M H_2SO_4 at different applied overpotentials (0 to -200 mV). The potential perturbation amplitude was 10 mA and the frequency ranged from 10 kHz to 10 mHz. The spectra shown in Fig. 2 are typical of the HER and are characterized by a high-frequency response (caused by the charge/discharge of the electrical double layer) and low-frequency response (related to the Faradaic reaction, i.e., the HER). The low-frequency semicircle decreases with increasing applied overpotential, corresponding to the rising HER current. An unusual feature of the spectra is the trend toward a diffusion response, which becomes apparent at frequencies below 1 Hz. This phenomenon has not yet been analyzed, as it seems to play a negligible role in the spectra.

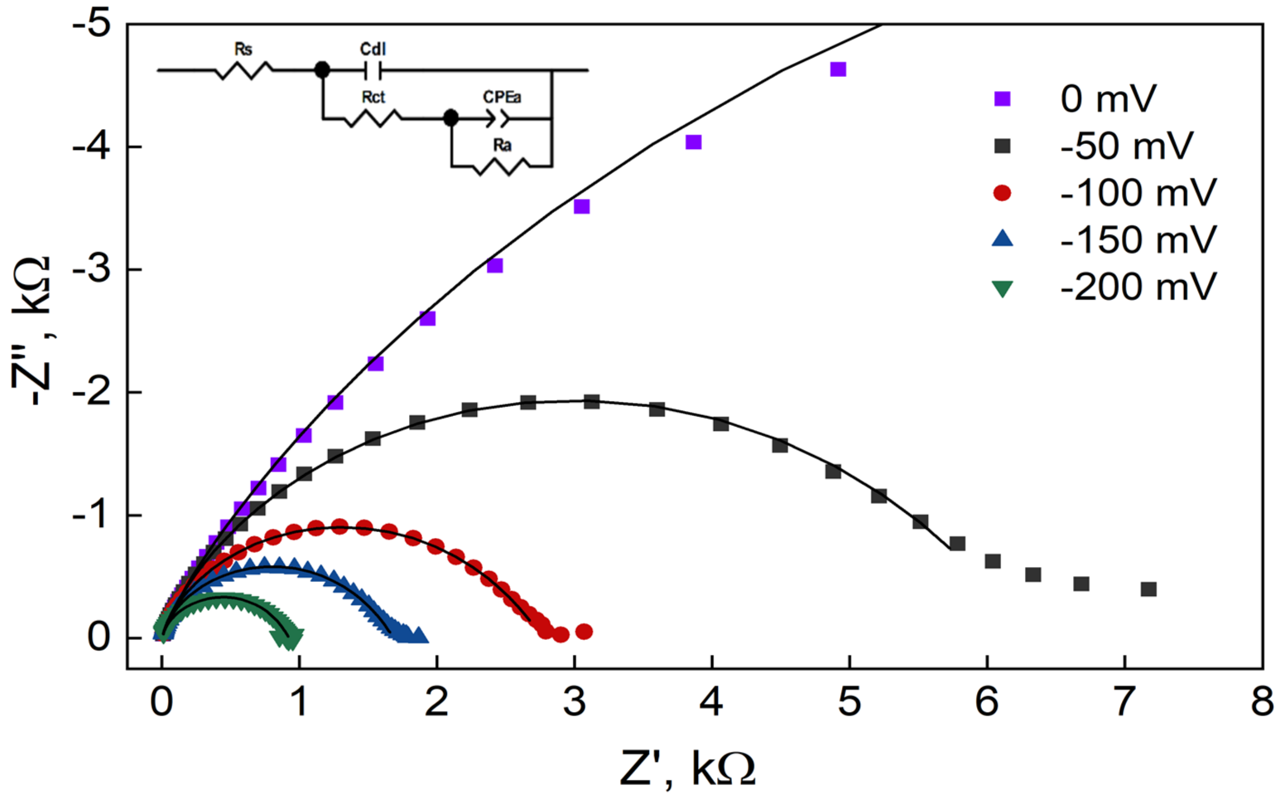

Fig. S9. *EIS spectra of the HER obtained on modified- nanosheets of Pt_3Te_4 induced by 0.3% H_2O_2-assisted sonication. The solid lines represent the equivalent circuit fit. The inset shows the equivalent circuit used for the fitting. In the Bode plots (not shown at the moment), it can be seen that the phase maximum was reached at very high frequencies (in the 10 – 1 kHz range), which is indicative of very fast hydrogen adsorption and relaxation kinetics. The electrodes exhibited blocking behavior, as the impedance magnitude reached a constant value at frequencies as high as 50 Hz.*

The relevant parameters of the equivalent circuit elements (R_s, C_{dl}, R_{ct}, C_a, and R_a) were obtained from the equivalent circuit fitting and are presented in Table S1. Here, the n parameter of the CPE_a element used to model the low-frequency semicircle related to the HER had values over 0.6, indicating a very inhomogeneous H_{ads} layer, which may be related to the catalyst distribution on the surface of the electrode. R_a is related to the hydrogen adsorption and desorption rates, and its exponential decrease corresponds to the logarithmic relationship between current and overpotential, as described by the Tafel equation.

Table S1. Equivalent circuit parameters.

n	R_s	C_{dl}	R_{ct}	CPE_a	n	R_a
**0**	7.331	4.89E-07	180.4	1.17E-05	0.600	24118
-0.05	6.777	4.66E-07	226.8	8.52E-06	0.628	6059
-0.10	6.634	4.55E-07	221.7	8.58E-06	0.624	2541
-0.15	6.987	4.20E-07	273.3	8.11E-06	0.637	1404
-0.20	7.413	3.94E-07	262.4	9.30E-06	0.653	655.5

S3. Oxide thickness estimation on the Pt_3Te_4 nanosheets

Equivalent oxide thicknesses were obtained from Pt 4f spectra by applying a Strohmeier-type overlayer model corrected with effective attenuation lengths (EALs). The oxidized Pt component (PtO_2-like, I_o) and the Pt_3Te_4 substrate components (Pt_2Te_2 plus $PtTe_2$ terminations, summed as I_m) were fit with shared constraints across potentials.

Thickness was computed as

$$d_{xps}(\mathrm{nm}) = \lambda_o \sin\theta \left(\frac{N_m \lambda_m I_o}{N_o \lambda_o I_m} + 1\right) \quad \text{(S1)}$$

where $\lambda_{m,o}$ are EALs at the Pt 4f kinetic energy (TPP-2M/NIST-style values) and N_m/N_o is the Pt atomic number-density ratio for Pt_3Te_4 vs PtO_2 (from compound densities and stoichiometry). Normal emission was used ($\sin\vartheta \approx 1$). Because oxidation is patchy, d_{xps} represents an equivalent thickness.

Table S2. Pt-oxide equivalent thickness d_{xps} for 0.2% H_2O_2-treated Pt_3Te_4 sample

Voltages	Surface oxide thickness [nm]
0 V	0.06 ±0.06
-0.2 V	0.24 ±0.13
-0.3 V	0.72 ±0.19
-0.4 V	1.16 ±0.23
-0.5 V	1.16 ±0.23
-0.6 V	1.17 ±0.23

S4. Comparison with other materials

Table S3. Comparison of the HER performances of Pt_3Te_4 with previously reported Pt-based and transition-metal dichalcogenides-based catalysts. NHCSs stand for nitrogen-doped hollow carbon spheres, NDs for nanodots.

Catalyst	Electrolyte	η, mV at −10 mA cm^{-2}	Tafel slope, mV dec^{-1}	Ref.
Commercial 20 wt% Pt/C (benchmark)	0.5 M H_2SO_4	~20–40 mV	≈30	[S1]
A/C-P-$PtTe_2$	0.5 M H_2SO_4	28	37	[S2]
P-$PtTe_2$	0.5 M H_2SO_4	57	88	[S2]
$PtTe_2$	0.5 M H_2SO_4	109	140	[S2]
$PtTe_2$ film	0.5 M H_2SO_4	330	85	[S3]
PtNi alloy confined in N-doped carbon (PtNi/NC)	0.5 M H_2SO_4	~35 mV	~30–40	[S4]
PtCo@PtSn	0.5 M H_2SO_4	21	26	[S5]
Pt@PtIr nanodendrites	0.5 M H_2SO_4	22	-	[S6]
Pt NDs	0.5 M H_2SO_4	26	-	[S6]
Pt/BNHCSs	0.5 M H_2SO_4	14		[S7]
Ru/BNHCSs	0.5 M H_2SO_4	43		[S7]

PtRu/BNC	0.5 M H_2SO_4	10		[S7]
Pt/C (20 wt%)	0.5 M H_2SO_4	12		[S7]
$PtTe_2$	0.5 M H_2SO_4	38.8	59.2	[S8]
$PtTe_2$	0.5 M H_2SO_4	540	110	[S9]
$PtSe_2$	0.5 M H_2SO_4	630	132	[S9]
PtS_2	0.5 M H_2SO_4	860	216	[S9]
Pt_3Te_4	0.5 M H_2SO_4	370	~150	[S10]
Pt_3Te_4	1 M KOH	678	~175	[S10]
VS_2 nanoflowers	0.5 M H_2SO_4	398	95	[S11]
WS_2 nanotriangles	0.5 M H_2SO_4	289	73	[S11]
MoS_2 nanotubes	0.5 M H_2SO_4	223	84	[S11]
MoS_2−MoP−Mo_2C@CW	0.5 M H_2SO_4	65	63	[S12]
N-Mo_2C-NDs@graphene	1 M KOH	84	74	[S13]
Ni_2P/Co_2P hollow nanoflowers	1 M KOH	153	61.75	[S14]
Pt_3Te_4 on $MoTe_2$	0.5 M H_2SO_4	39.6	32.7	[S15]
MoS_2	0.5 M H_2SO_4	136	73	[S16]
Pt_3Te_4	0.5 M H_2SO_4	46	36-49	[S17]
Pt_3Te_4	0.5 M H_2SO_4	113.1	51	This study

Pt_3Te_4 (0.3 % H_2O_2)	0.5 M H_2SO_4	78.7	53	This study

BIBLIOGRAPHY

S1. Wan, C.; Ling, Y.; Wang, S.; Pu, H.; Huang, Y.; Duan, X. Unraveling and Resolving the Inconsistencies in Tafel Analysis for Hydrogen Evolution Reactions. *ACS Cent. Sci.* **2024**, *10*, 658−665. https://doi.org/10.1021/acscentsci.3c01439

S2. Ma, H.; Huang, X.; Li, L.; Peng, W.; Lin, S.; Ding, Y.; Mai, L. Boosting the Hydrogen Evolution Reaction Performance of P-Doped $PtTe_2$ Nanocages via Spontaneous Defects Formation. *Nano-Micro Small* **2023**, *19*(41) 2302685. https://doi.org/10.1002/smll.202302685

S3. Mc Manus, J.B.; Horvath, D.V.; Browne, M.P.; Cullen, C.P.; Cunningham, G.; Hallam, T.; Zhussupbekov, K.; Mullarkey, D.; Coileain, C.O.; Shvets, I.V.; Pumera, M.; Duesberg, G.S.; McEvoy, N. Low-temperature synthesis and electrocatalytic application of large-area $PtTe_2$ thin films. *Nanotechnology* **2020**, *31*, 375601. https://doi.org/10.1088/1361-6528/ab9973

S4. Wang, X.; Che, J.; Wang, T.; Xu, F.; Duan, D. Nitrogen-doped carbon nanosheet confined PtNi alloy for efficient acidic electrocatalytic hydrogen evolution reaction. *Int. J. Hydrogen Energy* **2024**, *91*, 735-743. https://doi.org/10.1016/j.ijhydene.2024.10.193

S5. Chen, J.; Qian, G.; Zhang, H.; Feng, S.; Mo, Y.; Luo, L.; Yin, S. PtCo@PtSn Heterojunction with High Stability/Activity for pH-Universal H_2 Evolution. *Adv. Funct. Mater.* **2021**, *32*, 2107597. DOI: 10.1002/adfm.202107597

S6. Liu, C., Wei, Z., Cao, M., Cao, R. Pt nanodendrites with a PtIr alloy surface structure exhibit excellent stability toward acidic hydrogen evolution reaction. *Nano Res.* **17**, 4844–4849 (2024). https://doi.org/10.1007/s12274-024-6454-3

S7. Zhao, X.; Jiang, Y.; Wang, D.; Zhang, Y.; Chen, M.; Hu, G.; Zhang, H.; Jin, Z.; Zhou, Y. PtRu Intra-Cluster Electron Modulation Accelerates Multi-Scenario Hydrogen Evolution Reaction. *Adv. Energy Mater*. **2024**, *15*, 2305828. https://doi.org/10.1002/aenm.202405828

S8. Fu, J.; Peng, Y.; Zhou, L.; Wang, J.; Huan, Y.; Zhou, T.; Ding, H.; Wang, R.; Zhang, Y. Controllable growth of two dimensional stereoscopic $PtTe_2$ nanosheets for efficient electrocatalytic hydrogen evolution. *Chem. Commun*. **2025**, *61*, 18360-18363, https://doi.org/10.1039/D5CC05037E

S9. Chia, X.; Adriano, A.; Lazar, P.; Sofer, Z.; Luxa, J.; Pumera, M. Layered Platinum Dichalcogenides (PtS_2, $PtSe_2$, and $PtTe_2$) Electrocatalysis: Monotonic Dependence on the Chalcogen Size. *Adv. Funct. Mater*. **2016**, *26*(24), 4306-4318, DOI: 10.1002/adfm.201505402

S10. Supriya, S.; Antonatos, N.; Luxa, J.; Gusmão, R.; Sofer, Z. Comparison between layered Pt_3Te_4 and $PtTe_2$ for electrocatalytic reduction reactions. *FlatChem* **2021**, *29*, 100280. DOI: https://doi.org/10.1016/j.flatc.2021.100280

S11. Kadam, S. R.; Krishnappa, M.; Ghosh, S.; Sreedhara, M. B.; Neyman, A.; Upcher, A.; Nativ Roth, E.; Houben, L.; Zak, A.; Enyashin, A. N.; et al. Nanotubes and other nanostructures of VS_2, WS_2, and MoS_2: Structural effects on the hydrogen evolution reaction. *Appl. Mater. Today* **2024**, *39*, 102288. https://doi.org/10.1016/j.apmt.2024.102288

S12. Du, Q.; Wang, Y.; Fan, X.; Li, X.; Liu, Y.; Jiao, P.; Xing, P.; Yin, R.; Gan, W. Carbonized Wood Decorated with Ternary Heterogeneous MoS_2-MoP-Mo_2C Nanoparticles for pH-Universal Hydrogen Evolution. *ACS Appl. Mater. Interfaces* **2025**, *17* (9), 14025–14035. DOI: 10.1021/acsami.4c21484

S13. Liang, N.; Xu, H.; Zhang, H.; Zhang, Z.; Wang, M.; Jin, Z. Enhancing catalytic activity in Mo_2C nanodots via nitrogen doping and graphene integration for efficient hydrogen evolution under alkaline conditions. *J. Colloid Interf. Sci*. **2025**, *684*, 1-9. https://doi.org/10.1016/j.jcis.2025.01.073

S14. Xu, B.; Duan, M.; Shen, K.; Guo, X.; Yang, X.; Zhang, M.; Yue, B.; Zhang, M.; Zhang, J.; Jin, Z. *ACS Appl. Mater. Interfaces* **2024**, *16*(13), 16399–16407. https://doi.org/10.1021/acsami.4c02566

S15. Bae, D.; Park, K.; Kwon, H.; Won, D.; Ling, N.; Baik, H.; Yang, J.; Park, H. J.; Cho, J.; Yang, H. Mitrofanovite, Layered Platinum Telluride, for Active Hydrogen Evolution. *ACS Appl. Mater. Interfaces* **2021**, *13* (2), 2437–2446. DOI: 10.1021/acsami.0c16098

S16. Xia, H.; Shi, Z.; Gong, C.; He, Y. Recent strategies for activating the basal planes of transition metal dichalcogenides towards hydrogen production. *J. Mater. Chem. A* **2022**, *10* (37), 19067–19089.

S17. Boukhvalov, D. W.; Cheng, J.; D'Olimpio, G.; Bocquet, F. C.; Kuo, C.-N.; Sarkar, A. B.; Ghosh, B.; Vobornik, I.; Fujii, J.; Hsu, K.; et al. Unveiling the Mechanisms Ruling the Efficient Hydrogen Evolution Reaction with Mitrofanovite Pt_3Te_4. *J. Phys. Chem. Lett*. **2021**, *12* (35), 8627–8636. DOI: 10.1021/acs.jpclett.1c01261
